# Host Iron Withholding Demands Siderophore Utilization for *Candida glabrata* to Survive Macrophage Killing

**DOI:** 10.1371/journal.ppat.1001322

**Published:** 2011-03-17

**Authors:** Tracy Nevitt, Dennis J. Thiele

**Affiliations:** Department of Pharmacology and Cancer Biology, Duke University Medical Center, Durham, North Carolina, United States of America; Washington University School of Medicine, United States of America

## Abstract

The fungal pathogen *Candida glabrata* has risen from an innocuous commensal to a major human pathogen that causes life-threatening infections with an associated mortality rate of up to 50%. The dramatic rise in the number of immunocompromised individuals from HIV infection, tuberculosis, and as a result of immunosuppressive regimens in cancer treatment and transplant interventions have created a new and hitherto unchartered niche for the proliferation of *C. glabrata*. Iron acquisition is a known microbial virulence determinant and human diseases of iron overload have been found to correlate with increased bacterial burden. Given that more than 2 billion people worldwide suffer from iron deficiency and that iron overload is one of the most common single-gene inherited diseases, it is important to understand whether host iron status may influence *C. glabrata* infectious disease progression. Here we identify Sit1 as the sole siderophore-iron transporter in *C. glabrata* and demonstrate that siderophore-mediated iron acquisition is critical for enhancing *C. glabrata* survival to the microbicidal activities of macrophages. Within the Sit1 transporter, we identify a conserved extracellular SIderophore Transporter Domain (SITD) that is critical for siderophore-mediated ability of *C. glabrata* to resist macrophage killing. Using macrophage models of human iron overload disease, we demonstrate that *C. glabrata* senses altered iron levels within the phagosomal compartment. Moreover, Sit1 functions as a determinant for *C. glabrata* to survive macrophage killing in a manner that is dependent on macrophage iron status. These studies suggest that host iron status is a modifier of infectious disease that modulates the dependence on distinct mechanisms of microbial Fe acquisition.

## Author Summary

*Candida glabrata* is a major human pathogen due to its low susceptibility to conventional antifungal drugs and the dramatic increase in the number of immunocompromised individuals suffering from HIV AIDS, cancer, and diabetes. Iron overload is one of the most common genetically inherited diseases and reports suggest increased susceptibility of these patients to bacterial infection. The ability of microorganisms to obtain iron from their environment is a major determinant in their fitness and hence in their ability to cause infectious disease. Here we demonstrate that the siderophore iron carrier is critical for *C. glabrata* survival after ingestion by mouse and human macrophage immune effector cells. Through the generation of macrophage models of human iron overload disease we demonstrate that ingested *C. glabrata* cells sense altered macrophage iron levels, and that the Sit1 siderophore-iron transporter functions as a critical determinant in the ability of *C. glabrata* to survive macrophage killing in a manner that is dependent on macrophage iron status. Our results reveal a role for siderophore-iron as a source of iron during *C. glabrata* infection, suggest additional therapeutic intervention strategies, and support a pivotal contribution for a common human iron overload disease in the mechanisms used for Fe acquisition in *C. glabrata*.

## Introduction

*Candida glabrata* has emerged as an opportunistic fungal pathogen that causes life-threatening infectious disease in humans [1]. The dramatic rise in the number of immunocompromised individuals due to HIV infection and tuberculosis, and as a result of immunosuppressive regimens in cancer treatment and transplant interventions, have provided fertile ground for unchecked *C. glabrata* proliferation. While *Candida* species now account for over 10% of all bloodstream infections [2], the poor susceptibility of *C. glabrata* to antifungal therapeutics is in great part responsible for the high mortality rate of up to 50% associated with *C. glabrata* candidemia [3], [4]. The limited knowledge of virulence factors that contribute to the pathogenesis of *C. glabrata* demands insights into the biology of this opportunistic pathogen that contribute towards the successful colonization of the mammalian host.

Iron (Fe) is an essential metal for virtually all organisms. The ability of Fe to cycle between the reduced ferrous (Fe^2+^) and oxidized ferric (Fe^3+^) forms endows it with redox versatility that is utilized in both catalysis and structural biology. However, Fe^2+^ also has the potential to generate damaging reactive oxygen species (ROS) via Fenton/Haber Weiss chemistry [5] and, as a consequence, organisms have evolved sophisticated homeostatic mechanisms to tightly regulate the acquisition, utilization, storage and mobilization of Fe [6], [7].

Eukaryotic Fe homeostasis has best been described in the budding yeast, *Saccharomyces cerevisiae* that possesses two high-affinity mechanisms of Fe acquisition [7]. In an aerobic environment, Fe is oxidized and largely insoluble [8] and one mechanism relies on the reduction of environmental Fe sources by cell surface reductases and transport through a ferroxidase/permease complex composed of the Fet3 and Ftr1 proteins, respectively. The activity of the Fet3 Cu-dependent ferroxidase is essential for Fe transport through Ftr1 and, thus, reductive high-affinity Fe assimilation is dependent on adequate cellular Cu bioavailability. Alternatively, *S. cerevisiae* expresses cell surface siderophore transporters. Synthesized and secreted by most bacteria and fungi as well as some higher plants, siderophores are low molecular weight organic chelators with high affinity for Fe^3+^ (binding constants between 10^23^ M and 10^49^ M). Given the low Fe bioavailability in an aqueous environment (free Fe^3+^ concentration below 10^−9^ M at pH 7) and the further strict active withholding of Fe within mammalian hosts (free serum Fe concentration of approximately 10^−24^ M) [9], siderophores represent a powerful and widespread mechanism for microbial Fe scavenging. This is underscored by the fact that bacteria and fungi have evolved transporters that allow them to utilize siderophores they themselves do not produce (xenosiderophores) and several fungi, such as *Candida* species, and bacteria do not possess the ability to synthesize siderophores yet express transporters for xenosiderophores [7]. Although the mechanisms of Fe homeostasis in *Candida albicans*, similar to most fungi, differ significantly from that of *S. cerevisiae* and *C. glabrata* [10], iron-regulated CaSit1 shares high homology with *S. cerevisiae* siderophore transporters and its deletion compromises utilization of fungal ferrichrome-type hydroxamate siderophores. CaSit1 has been shown to be required for the invasion and penetration of human epithelial cells in an in vitro model of oral candidiasis but was not required for murine systemic *candida* dissemination [11], possibly reflecting the sterile environment of the serum in which siderophore concentrations are predicted to be negligible. The absence of an identifiable heme receptor in *C. glabrata* present in *Candida albicans* [12], suggests that *C. glabrata* may rely predominantly on the solubilization of the circulating exchangeable Fe pool to meet its requirements for Fe. Given that mammals are host to a formidable number of microbial species, the aggressive exploitation of local siderophore reservoirs that allows for high-affinity Fe uptake from virtually any host ligand is likely to play a critical role in microbial virulence.

Mammalian cellular immunity relies heavily on the action of macrophages, cells that also play a critical role in systemic Fe homeostasis [13]. A key mechanism through which macrophages mediate microbial containment is through the localized reduction of serum transferrin-bound Fe, decreased Fe export and the depletion of Fe from the macrophage phagosome, an intracellular compartment that surrounds ingested microorganims. These macrophage activities promote the killing of microorganisms by depriving them of Fe essential not only for cellular biochemistry but also for incorporation into microbial ROS-detoxifying enzymes, such as catalase or superoxide dismutase, that are critical to resistance to high levels of oxidative stress generated in the phagosome [14]. Emerging evidence for increased infections in patients with Fe overload disease highlights the strict requirement for tight host Fe restriction in microbial containment [15]. Hereditary hemochromatosis (HH) is the most common single-gene disorder affecting Caucasian individuals of European descent [16]. Mutations associated with HH are found in the gene encoding the Fe exporter, ferroportin (Fpn), in *HAMP*, encoding the peptide hormone hepcidin and negative regulator of Fpn, as well as in genes encoding regulators of *HAMP* expression. Hepcidin expression and release from the liver leads to targeted Fpn degradation at a systemic level resulting in a block in dietary Fe uptake through enterocytes and a block in Fe release from macrophage and hepatocyte stores [17]. Although several of the mutations that underlie HH lead to similar outcomes of pathophysiological Fe overload in parenchymal organs, the clinical presentations differ with regard to macrophage Fe levels which are low when hepcidin levels are low or fails to be recognized by Fpn, and high when hepcidin levels increase or Fpn Fe-export activity is compromised. Given the critical role of macrophages in immunity, the dominant effects of Fpn activity on macrophage Fe status may be predicted to impinge on their immuno-protective functions.

Here we describe the *C. glabrata* Sit1 siderophore transporter, its regulation under Fe deficiency and substrate siderophore specificity. In infection assays using both mouse and human macrophage cell lines we observe a strong Sit1-mediated, siderophore-dependent increase in *C. glabrata* survival that is blunted with increased macrophage Fe levels. Genetic manipulation of macrophage Fe status to parallel that which is observed in human diseases of Fe overload revealed an inverse correlation between macrophage Fe and *C. glabrata* dependence on siderophore-Fe as indicated by yeast survival rates as well as by *SIT1* expression in the macrophage phagosome. Consistent with this, infection of primary macrophages derived from a mouse model of Ferroportin disease in which macrophage Fe levels are chronically elevated demonstrated enhanced *C. glabrata* survival that is independent of Sit1-mediated siderophore utilization. These observations are consistent with reports of greater microbial burden in patients with Fe overload disease [15], [18], [19], [20], [21] and predict that mutations affecting macrophage Fe levels may not only compromise microbial containment, but may also dictate the dependence of microbes on distinct mechanisms of Fe uptake.

## Results

### The *Candida glabrata* genome encodes a single siderophore transporter

**Figure 1:**
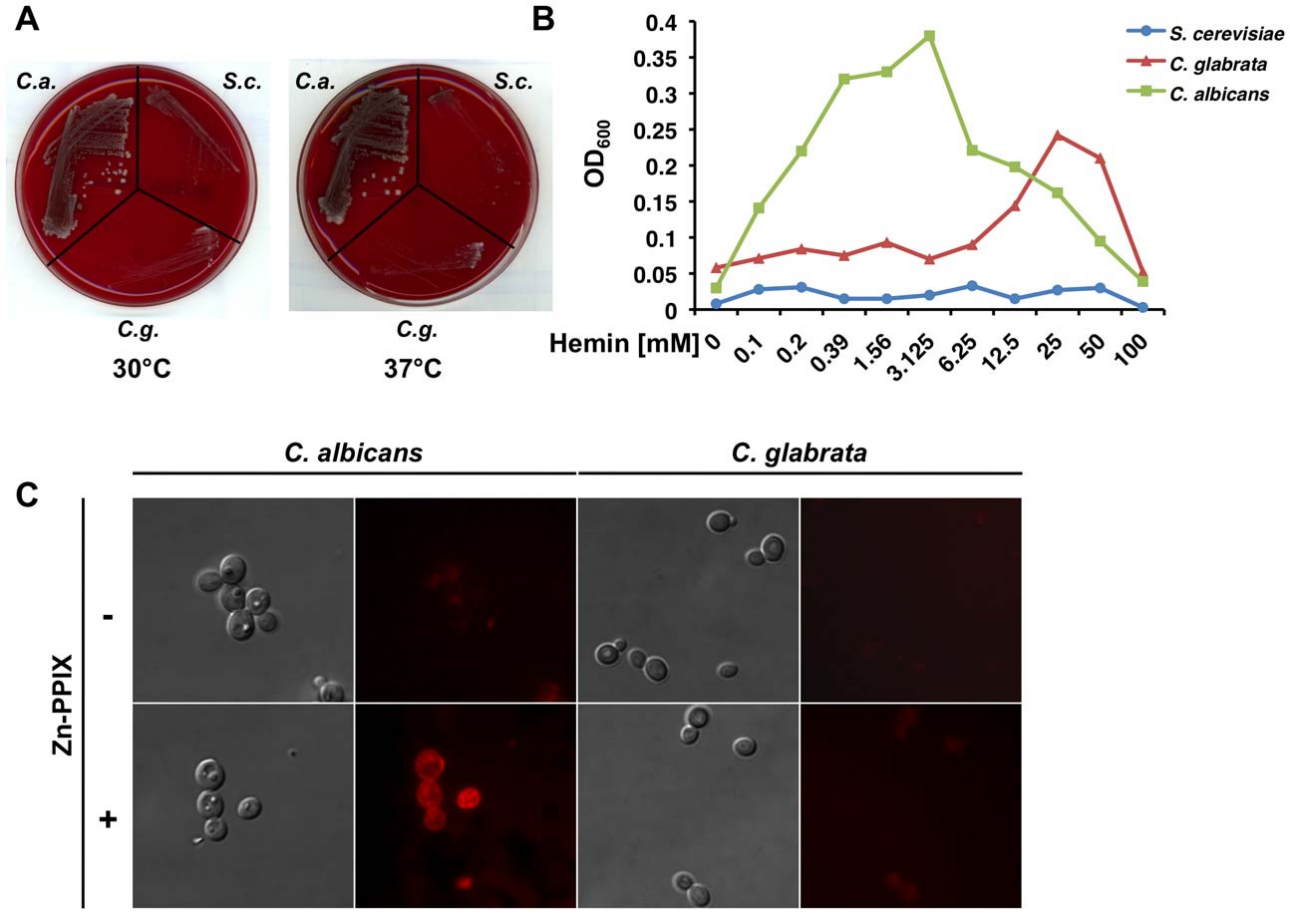
Heme is an inefficient source of Fe for *C. Glabrata*. **A** Hemolytic activity of *Candida albicans*, *C. glabrata* and *S. cerevisiae*. Wild type strains were streaked onto 5% blood sheep agar plates and grown at 30 °C or 37 °C with 5% CO_2_ for 48–72 hours. **B** Heme does not efficiently promote the growth of *C. glabrata* under Fe deficiency. *Candida albicans*, *C. glabrata* and *S. cerevisiae* were grown in Fe-deficient medium supplemented with the indicated concentrations of hemin and growth determined after 12 hours. **C** *Candida albicans* and *C. glabrata* were grown under Fe deficiency supplemented or not with 20 µM Zn-PPIX for 3 hours and observed by live fluorescence microscopy.

Fe acquisition is a critical microbial virulence determinant due to the Fe-restricted nature of the mammalian host. The great majority of body Fe is compartmentalized within red blood cells bound as heme to hemoglobin. Several microorganisms, including *C. albicans*, can access this rich Fe source by secreting hemolytic factors that results in red blood cell lysis [22]. Heme can then be taken up, often by means of a dedicated receptor (such as Rbt5 and Rbt51 in *C. albicans* [12]) and the porphyrin ring hydrolysed by intracellular heme oxidases [23]. To test whether red blood cells represent an efficient source of Fe for *C. glabrata*, we grew the wild type strain as well as that of *C. albicans* and *S. cerevisiae*, on sheep blood agar plates containing 5% whole blood. Formation of a hemolytic halo surrounding yeast colonies was evaluated after 48–72 hours growth at 30 °C or 37 °C with 5% CO_2_. Unlike the robust growth and associated hemolytic activity observed for *C. albicans*, *C. glabrata* and *S. cerevisiae* displayed very weak hemolysis when grown on blood sheep agar (**Figure 1A**). Furthermore, whereas *C. albicans* was able to efficiently utilize heme as a source of Fe at sub-micromolar hemin concentrations, *C. glabrata* required 100-fold higher hemin concentrations to achieve the same level of growth. In the same way, higher hemin concentrations were required for a slower growth of *C. glabrata* due to hemin toxicity when compared to *C. albicans* (**Figure 1B**). The observed differences in growth when heme is the sole Fe source may due to the absence of an identifiable heme receptor in the *C. glabrata* genome that is present in *C. albicans*. To address this possibility, C. *albicans* and *C. glabrata* were cultured in Fe-deficient medium supplemented or not with 20 µM zinc protoporphyrin (ZPP) for 3 hours. In contrast to heme, which is non-fluorescent due to the quenching of the intrinsic porphyrin fluorescence by Fe, ZPP fluoresces and its cellular uptake can be evaluated by in vivo microscopy. **Figure 1C** shows that in contrast to *C. albicans* that showed intense intracellular fluorescence, this was not observed for *C. glabrata,* the intracellular fluorescence of which did not rise above background as determined by comparison to the signal obtained by growth under Fe deficiency alone. This suggests that *C. glabrata* is not capable of taking up heme. Thus, given its weak hemolytic activity and inability to efficiently import heme, we predict that *C. glabrata* relies primarily on circulating host Fe sources to satisfy its requirements for Fe.

**Figure 2:**
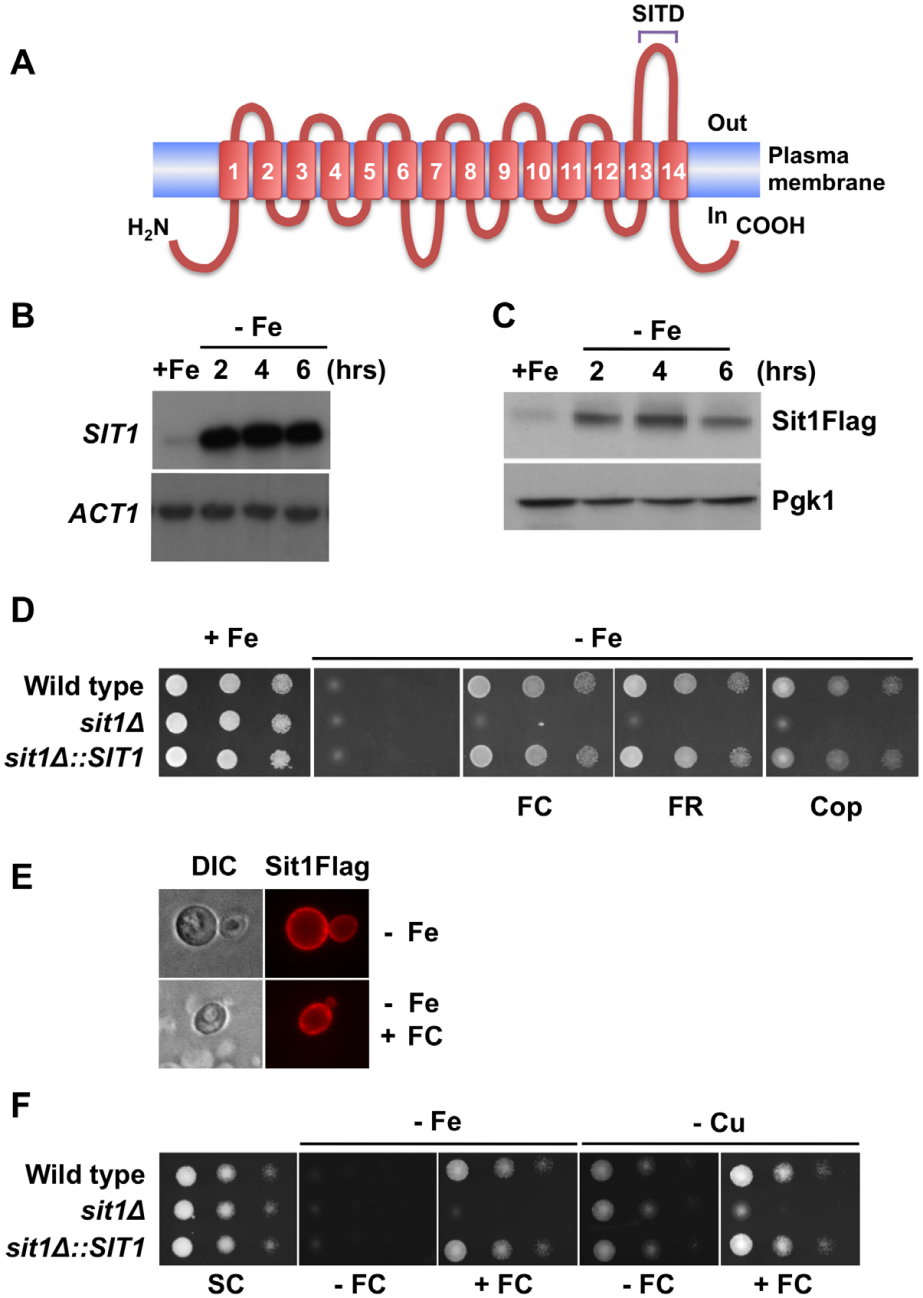
*SIT1 substrate specificity and regulation under Fe deficiency*. **A** Schematic illustration of the predicted Sit1 topological features. Blast analyses of the carboxyl-terminal loop identified this conserved region as a SIderophore Transporter Domain (SITD). **B** RNA blot showing elevated steady-state levels of *SIT1* mRNA under Fe deficiency at the time points indicated after chelation of extracellular Fe with BPS. **C.** Accumulation of Sit1Flag protein levels at the indicated time points following the addition of BPS. **D** Spot assays showing compromised growth of yeast strains under Fe limitation in the absence of substrate siderophores. All siderophores were used at a concentration of 10 µM and BPS was added at a concentration of 100 µM. **E** Sit1 is localized to the plasma membrane under Fe deficiency. Indirect immunofluorescence microscopy showing the accumulation of Sit1Flag at the plasma membrane under Fe deficiency in the presence or absence of ferrichrome (FC). Ferrichrome was added to Fe-deficient yeast cells 20–30 minutes prior to microscopic visualization **F** Siderophore utilization is functional under copper (Cu) deficiency. Spot assays were performed under either Fe limitation or Cu limitation in the presence or absence of ferrichrome as indicated.

Analysis of the *Candida glabrata* genome [24] predicts the existence of orthologues to the *Saccharomyces cerevisiae* high affinity reductive Fe assimilation pathway as well as a single open reading frame, *CAGL0E04092g*, encoding a predicted membrane protein with 14 transmembrane domains and a high degree of identity (72%) to the siderophore-Fe transporter, Arn1, that we have designated *SIT1* (Siderophore Iron Transporter 1) (**Figure 2A** and **Figure S1A**). Computational topological analyses of Sit1 identified sequence signatures characteristic of members of the Major Facilitator Superfamily of transporters (data not shown). In addition, Sit1 orthologues predicted to exist across the ascomycetes, and to lesser extent basidiomycetes, share high sequence identity within the carboxyl terminus of the protein. Protein BLAST analysis [25] of a 51 amino acid sequence comprising the last extra-cellular loop (from amino acid residue 531 to 581) exclusively returned known siderophore transporters and uncharacterized proteins predicted to encode fungal siderophore transporters (data not shown). This SIderophore Transporter Domain (SITD) is conserved in many pathogenic fungi (**Figure 2A**).

### Sit1 mediates siderophore-Fe utilization under conditions of Fe deficiency

To investigate the effects of Fe availability on *SIT1* expression, *C. glabrata* was grown under conditions of Fe-repletion or Fe-deficiency. **Figure 2B** shows that steady state *SIT1* mRNA levels are elevated under Fe deficiency and that this elevation is sustained throughout the time-course of the experiment over 6 hours. To analyze the levels of Sit1 protein as a function of Fe availability, a Flag epitope was introduced at the carboxyl terminus of *SIT1* and the fusion gene, encoding a functional protein, was integrated at the endogenous *SIT1* genomic locus (**Figure S1B**). Similar to what was observed for *SIT1* mRNA, Sit1Flag fusion protein expression is low under Fe repletion but robustly accumulates under conditions of Fe deficiency (**Figure 2C**).

Siderophore production is common among most microorganisms and is a major mechanism of Fe solubilization and acquisition. The very high Fe-binding constants observed for siderophores of fungal origin (approximately 10^30^ M at pH 7) are several orders of magnitude higher than that of the Fe^2+^ chelator BPS, which inhibits the Fet3/Ftr1 pathway of Fe^2+^ uptake. We reasoned that if a siderophore biosynthetic pathway exists in this microorganism, *C. glabrata* would exhibit growth in the presence of BPS. As shown in **Figure 2D**, growth of the wild type strain was severely compromised in Fe-deficient medium chelated with 100 µM BPS. This is consistent with a predicted lack of siderophore biosynthetic machinery in *C. glabrata* based on the absence of identifiable orthologues to the gene encoding ornithine-N^5^-oxygenase that catalyses the first committed step in fungal siderophore biosynthesis (the DNA and protein sequences of the *Aspergillus nidulans* ornithine-N^5^-oxygenase SidA were used to perform the BLAST analyses) [26]. To address the role of the Sit1 transporter in xenosiderophore utilization, a mutant lacking the *SIT1* gene, as well as the reconstituted strain in which the wild type *SIT1* gene was integrated in the *sit1*Δ strain at the endogenous genomic locus, were generated and evaluated for growth in Fe-deficient medium alone or in the presence of several structurally distinct fungal and bacterial siderophore substrates. Supplementation of Fe-deficient media with the fungal hydroxamate-type siderophores ferrichrome, ferrirubin or coprogen promoted robust growth of the wild type and *SIT1*-reconstituted strains (**Figure 2D**). In contrast, the *sit1*Δ mutant strain was unable to use siderophore-Fe, strongly supporting the notion that Sit1 is a siderophore transporter capable of utilizing these xenosiderophores. Neither the ester-linked siderophore triacylfusarine C, nor the bacterially-derived siderophores enterobactin, salmochelin, yersiniabactin or desferrioxamine were able to complement the growth of *C. glabrata* under iron deficiency (**Figure S1C**) suggesting that these siderophores are either not transported by Sit1 or *C. glabrata* lacks a mechanism of releasing the Fe from these siderophores intracellularly.

The subcellular localization of Sit1 was evaluated using the Sit1Flag and Sit1mCherry fusion proteins. Previous studies in *S. cerevisiae* have shown that during Fe deficiency the Arn1 siderophore transporter is not localized to the plasma membrane but rather to intracellular endosomal vesicles [27]. Trafficking of Arn1 to the plasma membrane is a substrate-dependent process that occurs in a dose-dependent manner [28], [29]. In contrast to this, indirect immunofluorescence as well as live microscopy of *C. glabata* Sit1 revealed strong accumulation of the Sit1 protein at the plasma membrane under Fe deficiency in the absence of siderophore substrate and this accumulation was largely unaltered by the addition of ferrichrome (**Figure 2E** **and Figure S1D**).

### Siderophore utilization is functional under Cu deficiency

High-affinity elemental Fe uptake requires the activity of the Fet3/Ftr1 ferroxidase/permease complex, with the protein complex assembled in the ER and 4 Cu ions loaded onto Fet3 within the Golgi. Under Cu deficiency, apo-Fet3-Ftr1 is assembled and targeted to the plasma membrane, but the absence of ferroxidase activity abolishes high-affinity Fe^2+^ transport through Ftr1 [30]. As such, elemental Fe uptake is a copper-dependent mechanism. In contrast, siderophore transporters are not known to require Cu for activity and would be predicted to be functional under low Cu conditions. **Figure 2F** shows that siderophore utilization is functional under Cu deficiency as imposed by growth in the presence of the Cu chelator BCS suggesting that Fe-siderophore transport is likely to be critical as the sole mechanism of high affinity Fe acquisition under conditions of Cu deficiency.

### Sit1-mediated siderophore utilization enhances the survival of *C. glabrata* to macrophage killing

**Figure 3:**
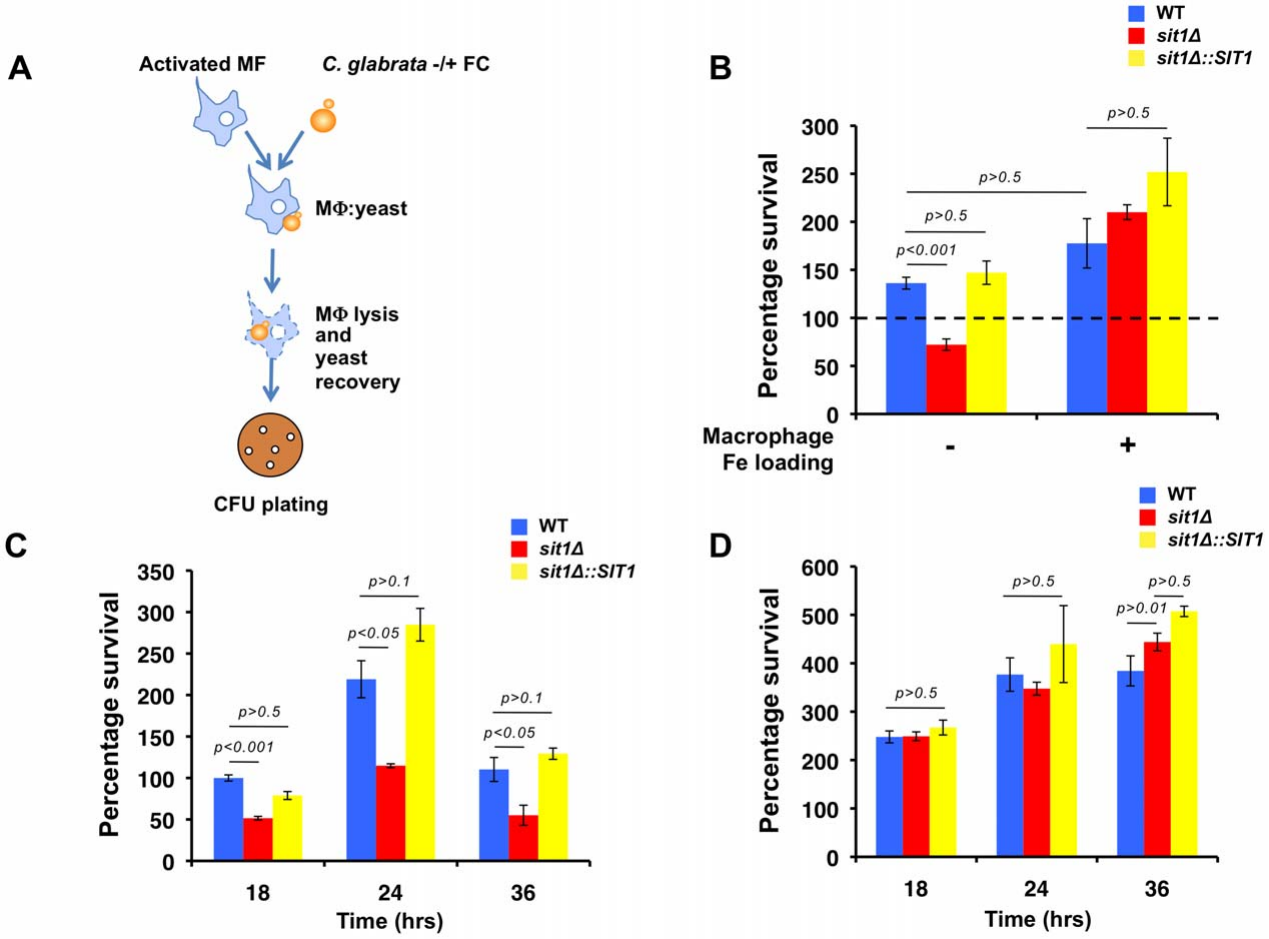
Macrophage Fe status determines the degree of Sit1 dependence for *C. glabrata* to survive macrophage killing. **A** Schematic flowchart of the macrophage infection assay developed for *C. glabrata* virulence studies. *C. glabrata* cells were grown in low Fe medium for 5 hours and pulsed with 10 µM ferrichrome (FC) for 30–45 minutes prior to exposure to activated mouse macrophages. *C. glabrata* cells were recovered from the lysed macrophages, plated on YPD and colony forming units (CFU) counted after 2 days at 30 °C. Data were analyzed with use of one-way ANOVA. **B** Sit1-mediated siderophore utilization enhances the survival of *C. glabrata* to macrophage killing activities (left columns) and this is ameliorated by elevated intracellular macrophage Fe. Macrophages were Fe-loaded with ferric ammonium citrate [20 µM] for 2 days prior to infection with *C. glabrata*. Each experiment included 6 replicates per experimental sample. Results are shown as mean survival compared to the wild type grown in the absence of ferrichrome supplementation (dashed line) and are representative of at least 3 experiments. Bars represent standard error. **C** The human monocytic cell line, U937, was differentiated into mature macrophages prior to infection with the indicated *C. glabrata* strains grown under Fe deficiency without or with supplementation of 10 µM FC and yeast survival evaluated after 18, 24 and 36 hours of infection. The results depicted show the mean survival of the wild type, *sit1*Δ and *sit1*Δ*::SIT1* strains grown in the presence of FC prior to macrophage infection. Bars indicate standard error. **D** U937 macrophages were Fe-loaded with ferric ammonium citrate [20 µM] for 2 days prior to infection with *C. glabrata* strains grown under Fe deficiency without or with supplementation of 10 µM FC and yeast survival evaluated after 18, 24 and 36 hours of infection, as described above.

The ability of *C. glabrata* to exploit local xenosiderophore reservoirs in the absence of an endogenous siderophore biosynthetic pathway suggests a selective advantage imparted by this mechanism of Fe uptake that may impact on the survival of this fungal pathogen. Fe acquisition is challenging within the mammalian host, in which Fe is tightly bound to proteins and ligands in extracellular and intracellular environments. The further active depletion of Fe from the phagosomal compartment amplifies the microbicidal potency of macrophages as microorganisms defend themselves from ROS under conditions that are expected to compromise ROS-detoxifying enzyme activity. We tested whether Fe deficiency would increase the susceptibility with which *C. glabrata* resists macrophage killing and if this might be circumvented by exposure of *C. glabrata* to substrate siderophore. Using the mouse macrophage-like cell line, *J774A.1*, isogenic wild type, *sit1*Δ and *sit1*Δ*::SIT1 C. glabrata* strains were grown under Fe limiting conditions and briefly incubated in the absence or presence of ferrichrome prior to co-culture with activated macrophages (**Figure 3A**). *C. glabrata* cells were recovered by cell lysis and survival was evaluated by quantitating colony forming units (CFU) and comparing percentage yeast survival relative to the wild type strain grown under Fe deficiency (represented by a dashed line). **Figure S2A** shows that when *C. glabrata* cells were grown under Fe deficiency prior to macrophage infection, differences were not observed in the survival of the three strains to macrophage killing. However, growth of both the wild type and reconstituted *sit1*Δ*::SIT1* strains in the presence of ferrichrome significantly increased survival when compared to the *sit1*Δ strain, indicative of enhanced *C. glabrata* ability to survive macrophage killing as a result of siderophore-Fe utilization (**Figure 3B**, left columns). This suggests that Sit1-mediated ferrichrome utilization provides a survival advantage to phagocytosed *C. glabrata* cells that is not observed in the strain that cannot internalize this Fe source. To address whether the decreased survival of the *sit1*Δ strain could be attributed to altered levels of phagocytosis of the mutant yeast cells rather than decreased Fe satiety, CFU were monitored 2 hours post-infection and no significant differences between the wild type, *sit1*Δ and *sit1*Δ*::SIT1* strains were observed in the presence or absence of ferrichrome, strongly suggesting equally efficient phagocytosis of the three yeast strains by macrophages (**Figure S2A**).

To test whether the Sit1-dependent increase in *C. glabrata* survival in the presence of siderophore was a consequence of macrophage Fe status, macrophages were Fe-loaded prior to infection with the *C. glabrata* strains and the levels of ferritin, the primary intracellular Fe storage protein, were evaluated by immunoblotting. As shown in **Figure S2B**, activated macrophages cultured under Fe supplementation (lane 4) showed higher ferritin protein levels than those cultured in normal medium (lane 2). Under these conditions of exogenous Fe loading, differences in survival were not observed between the wild type, *sit1*Δ and *sit1*Δ*::SIT1* yeast strains grown in the presence or absence of ferrichrome (**Figure 3B**, right columns). Furthermore, significantly higher CFU were recovered from Fe-loaded macrophages under all conditions tested when compared to infection of macrophages growing in the absence of Fe supplementation, suggesting that elevated Fe compromises the ability of macrophages to contain *C. glabrata* proliferation within the phagosome. To ascertain whether Fe-loading compromises macrophage activation and the ability to mount an inflammatory response, secreted levels of TNF-alpha, an acute phase inflammatory cytokine secreted by activated macrophages, were assayed and no significant changes were observed between that secreted from activated Fe-loaded macrophages and activated macrophages cultured in standard growth media (**Figure S2C**).

Despite the fact that the overall mechanisms of innate and adaptive immunity are relatively conserved between mice and humans, there are striking immunological differences between the two species [31]. In particular, there is some controversy over whether human macrophages express functional inducible nitric oxide synthase (iNOS). This enzyme catalyzes the production of NO from L-arginine that, in the oxidative environment of the phagosome, reacts with superoxide leading to the generation of peroxynitrite, a powerful oxidant used by macrophages and neutrophils in microbial killing during the oxidative burst. Given this and other differences in mouse and human macrophage immunobiology, we asked whether Sit1-mediated siderophore utilization enhanced *C. glabrata* survival to killing by human macrophages. To test this, we used the U937 cell line derived from a hystiocystic lymphoma whose differentiation and activation have been previously described [32], [33]. Upon differentiation into mature macrophages, U937 cells were infected with the wild type, *sit1*Δ and *sit1*Δ*::SIT1 C. glabrata* strains grown under Fe deficiency, with or without a brief exposure to ferrichrome, and yeast CFU quantitated after 18, 24 and 36 hours of co-culture. **Figure 3C** shows that when exposed to ferrichrome, the wild type and *SIT1* reconstituted strains displayed enhanced survival at all time points in contrast to the *sit1*Δ mutant strain that is unable to transport the siderophore. Pre-loading U937 cells with Fe by culturing in the presence of 20 µM FAC promoted the growth of *C. glabrata* and abrogated the requirement for Sit1-mediated siderophore utlization in enhanced *C. glabrata* survival (**Figure 3D**). These results recapitulate the observations made in the *J744A.1* mouse macrophage cell line and suggest that Sit1-mediated siderophore utilization protects *C. glabrata* against the intracellular microbicidal activities of macrophages. Given the generation of ROS in the phagosome of activated macrophages (**Figure S2D**), indicative of higher levels of oxidative stress, we hypothesize that siderophore-Fe plays a role in providing the Fe required for DNA replication and cellular proliferation as well as for defence against oxidative stress. Moreover, these results indicate that macrophage Fe-withholding from phagocytosed microorganisms is fundamental for the containment of microbial growth and also impacts on the mechanisms available for microbial iron uptake, imposing a strict dependence of *C. glabrata* on the availability and utilization of xenosiderophores via Sit1.

### Macrophage Fe status dictates the dependence of *C. glabrata* on Sit1 function

**Figure 4:**
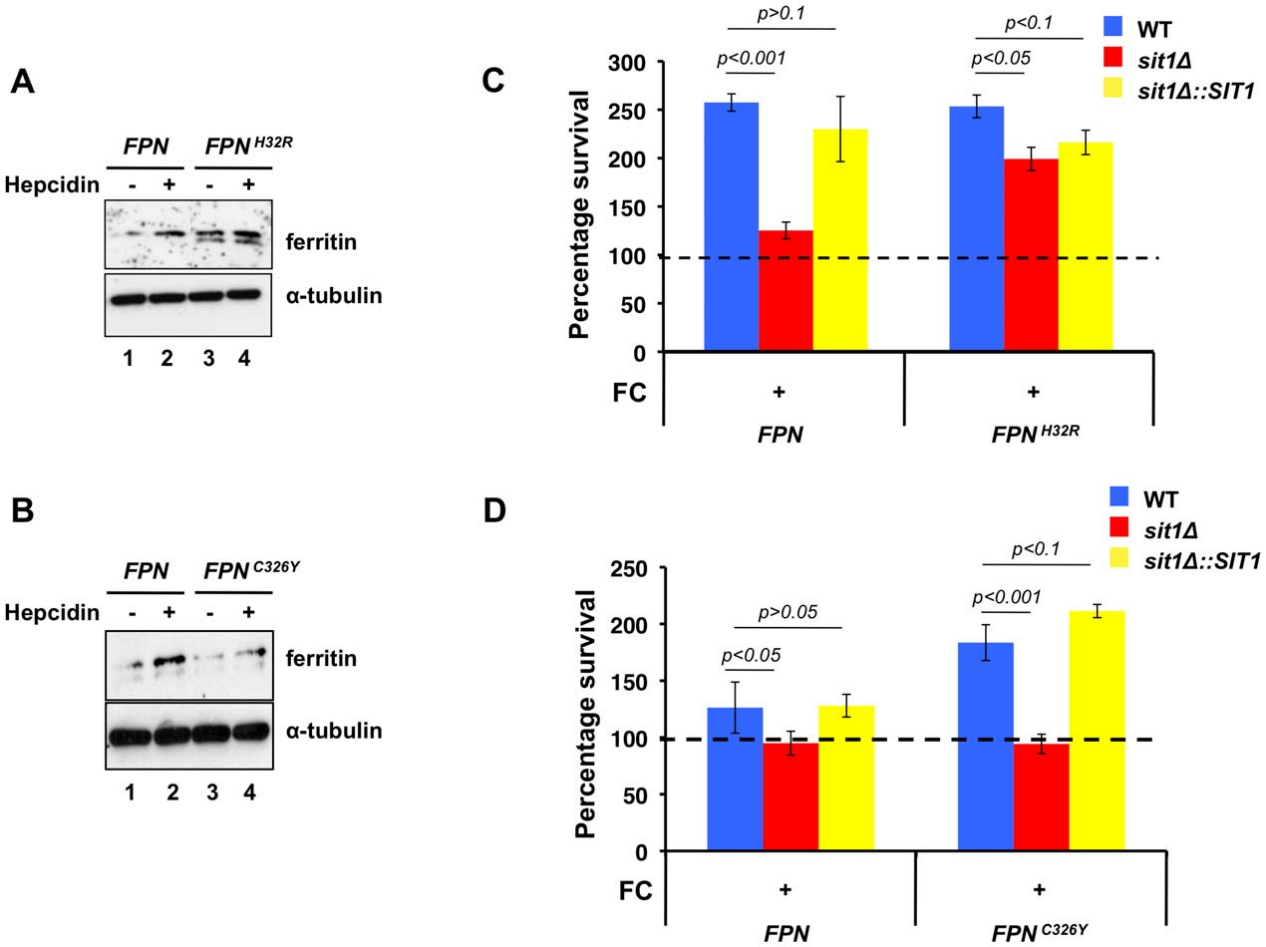
Macrophage models of Fe homeostasis disease modulate survival of *C. glabrata* and dependence on siderophore Fe. **A** *J774A.1* cells stably expressing *FPN^H32R^* are chronically Fe-loaded. *J774A.1* stably expressing either wild type *FPN* or *FPN^H32R^* were grown in the presence or absence of hecidin for 24 hours. Intracellular labile Fe levels were monitored by evaluating H- and L-ferritin levels through immunoblotting. **B** *J774A.1* cells expressing *FPN^C326Y^* are compromised for Fe accumulation in the presence of hepcidin. *J774A.1* stably expressing either wild type *FPN* or *FPN^C326Y^* were grown in the presence or absence of hepcidin for 24 hours. Intracellular Fe status was monitored by evaluating H- and L-ferritin levels. **C** Dependence on Sit1-mediated siderophore utilization for *C. glabrata* survival is reduced in macrophages expressing *FPN^H32R^*. Stable cell lines expressing the wild type and *FPN^H32R^* allele were infected with *C. glabrata* and CFU quantitated after 18–24 hours of co-culture. Results are shown as survival relative to wild type *C. glabrata* grown under Fe deficiency represented by a dashed line. Data were analyzed with use of one-way ANOVA. **D** Hepcidin insensitivity of the iron-deficient *FPN^C326Y^* allele imposes a greater dependence on siderophore-Fe when compared to the more Fe-loaded macrophages expressing the wild type *FPN* allele. Stable cell lines expressing the wild type and *FPN^C326Y^* allele grown in the presence of hepcidin were infected with *C. glabrata* and CFU counted. Results are shown as survival relative to wild type *C. glabrata* grown under Fe deficiency represented by a dashed line. Each experiment included 6 replicates per experimental sample. Results are shown as mean survival compared to the wild type grown in the absence of ferrichrome supplementation and are representative of at least 3 experiments. Bars represent the standard error. Data were analyzed with use of one-way ANOVA.

Based on the effect of macrophage Fe loading on *C. glabrata* Sit1-mediated siderophore-dependent survival, we asked whether mutations that alter macrophage Fe homeostasis might similarly modulate the dependence of *C. glabrata* on siderophore-Fe acquisition. The sole mammalian Fe exporter Fpn is expressed on the surface of enterocytes, macrophages, hepatocytes and placental cells where it plays a fundamental role in systemic Fe homeostasis. Type IV hemochromatosis, or Ferroportin disease, is an autosomal-dominant condition that arises from missense mutations in Fpn that compromise regulated Fe export activity. Two often contrasting clinical presentations observed in patients result from distinct mutations in Fpn that either abolish the hepcidin binding site or compromise Fe export, leading to differential effects on macrophage Fe levels [16], [34], [35], [36]. We modulated macrophage Fe status by stably expressing a cDNA encoding the *FPN^C326Y^* hepcidin-insensitive disease mutation, associated with low macrophage Fe levels, or the *FPN^H32R^* allele identified in the *flatiron* mouse that fails to localize to the plasma membrane, leading to Fe-overload. Using ferritin protein levels as a biomarker of macrophage Fe loading, macrophages expressing the *FPN^H32R^* transgene were chronically iron-loaded in the absence of hepcidin when compared to macrophages expressing the wild type gene due to the loss in ability to export cellular Fe (**Figure 4A**, lanes 1 and 3). Incubation with hepcidin increased ferritin levels in cells expressing wild type *FPN*, as would be predicted from Fpn internalization and degradation, resulting in increased cellular Fe accumulation (**Figure 4A**, lane 2). In contrast, hepcidin had only a mild effect on *FPN^H32R^*-expressing macrophages (**Figure 4A**, lane 4). Given that Fpn functions as a dimer [37], this modest increase in ferritin levels may reflect the contribution of hepcidin-sensitive endogenous wild type homodimers that correctly localize to the plasma membrane. In contrast, macrophages expressing the *FPN^C326Y^* transgene show lower than wild type ferritin levels when grown under Fe-replete conditions in the presence of hepcidin, consistent with a chronic Fe efflux under conditions that induce Fpn turnover of the wild type protein (**Figure 4B**, lanes 2 and 4). Indirect immunofluorescence in macrophages expressing the wild type and mutant *FPN* transgenes was performed to monitor protein localization. Both the wild type and Fpn^C326Y^-expressing cells, but not cells expressing Fpn^H32R^, show plasma membrane ferroportin accumulation in the absence of hepcidin (**Figure S3A**). Exposure to hepcidin leads to a predominantly intracellular localization of the wild type protein, whereas the hepcidin-insensitive Fpn^C326Y^ protein remains at the cell surface.

Using these macrophages with altered Fe homeostasis, we ascertained whether genetically programmed differences in macrophage Fe levels impinge on the dependence of *C. glabrata* on siderophore-Fe for resistance to macrophage killing activities. Macrophages stably expressing the wild type and mutant forms of *FPN* were infected with wild type, *sit1*Δ and the *SIT1*-reconstituted *C. glabrata* strains that had been grown under Fe deficiency and exposed or not to ferrichrome. As shown in **Figure 4C**, wild type and *SIT1*-reconstituted *C. glabrata* yeast cells, but not *sit1*Δ cells, showed enhanced survival to macrophages expressing wild type Fpn only when grown in the presence of ferrichrome prior to infection, as interpreted from the increased number of CFU when compared to the wild type *C. glabrata* strain grown in the absence of FC (dashed line set at 100). No differences were observed in the survival of the three *C. glabrata* strains grown in the absence of ferrichrome supplementation (data not shown). This recapitulates the results obtained in non-transfected *J774A.1* mouse macrophages and human differentiated U937 cells in the absence of Fe supplementation (**Figure 3B**, left columns). In contrast, siderophore-dependent differences in *C. glabrata* survival were not observed between the yeast strains recovered from the chronically Fe-loaded macrophages expressing the *FPN^H32R^* transgene, supporting our previous results using exogenously Fe-loaded macrophages (**Figure 3B**, right columns). This is consistent with the high levels of labile intracellular Fe in *FPN^H32R^* macrophages that elevate the abundance of ferritin and might be inefficiently withheld from *C. glabrata*. In contrast, *C. glabrata* recovered from *FPN^C326Y^* macrophages cultured in the presence of hepcidin exhibit increased dependence on siderophore-Fe utilization for survival when compared to those recovered from macrophages expressing wild type Fpn (**Figure 4D**). This is consistent with the refractility of Fpn^C326Y^ to hepcidin-stimulated degradation and Fe levels in these macrophages that are lower than wild type. To ascertain whether Fe overload or deficiency might compromise the ability of macrophages to mount an inflammatory response, the levels of TNF-alpha were quantified from the culture medium of activated macrophages expressing the *FPN*, *FPN^C326Y^* and *FPN^H32R^* transgenes and no difference was found between cell lines (**Figure S3B**). Taken together, these data suggest that elevated macrophage Fe accumulation increases Fe accessibility to internalized *C. glabrata*. This ameliorates the strict requirement for Sit1-mediated siderophore utilization observed in macrophages with limited intracellular Fe.

### Macrophage Fe status modulates the expression of *C. glabrata SIT1*

**Figure 5:**
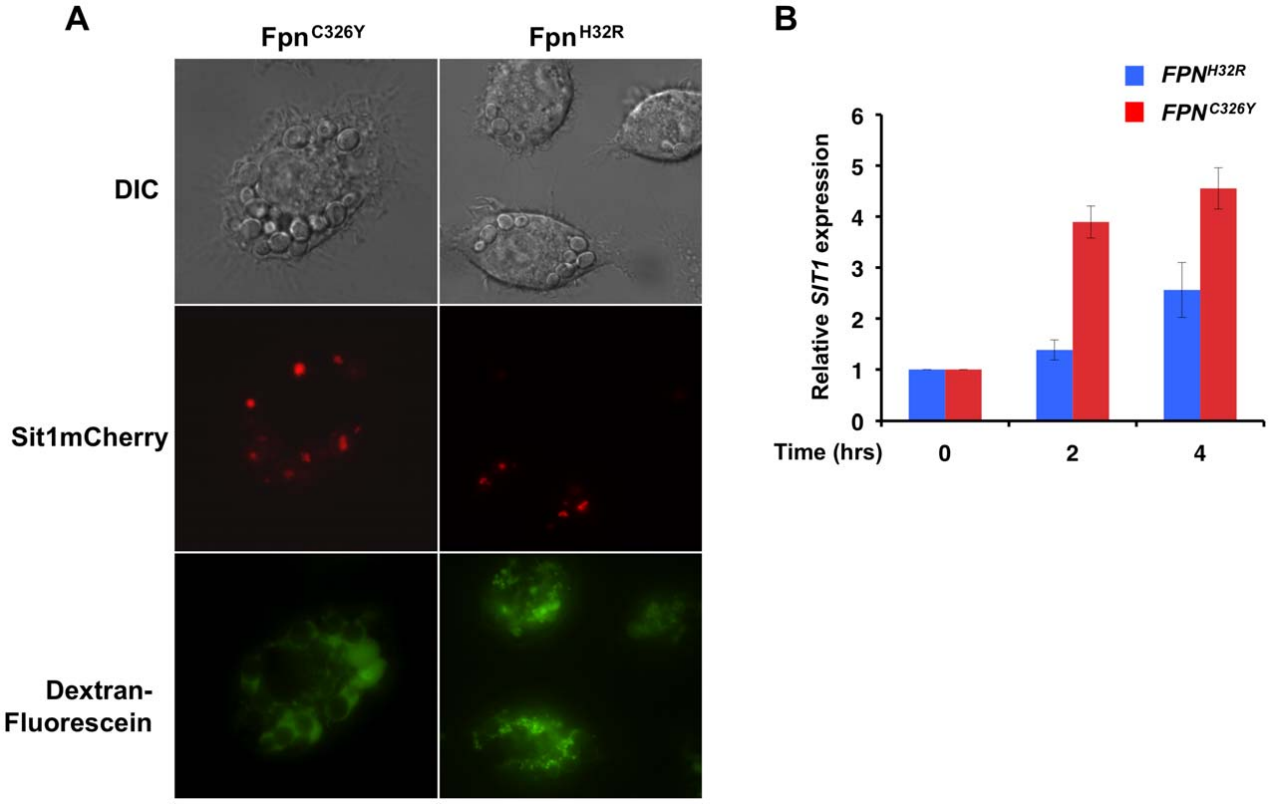
*C. glabrata* is responsive to host intracellular labile Fe pools. **A** *J774A.1* cells expressing the mutant *FPN* transgenes were infected with the *C. glabrata SIT1mCherry* strain reporter and visualized upon co-culture for 8 hours using live microscopy. Fluorescein-conjugated dextran beads were added prior to co-culture to ascertain whether yeast cells were located intracellularly rather than on the surface of macrophages. **B** Macrophage Fe overload is associated with increased *C. glabrata* Fe satiety whereas the severe macrophage Fe depletion associated with Fpn^C326Y^ leads to elevated *SIT1* expression. *C. glabrata* cells were recovered from infected macrophages at the indicated time points and the levels of *SIT1* mRNA quantified by RT-PCR. Each experiment included 3 replicates per experimental sample. Bars represent the standard error.

The results presented here suggest that Sit1-dependent siderophore utilization enhances *C. glabrata* survival to activated macrophages in a manner that is dependent on macrophage labile Fe status. *SIT1* characterization demonstrated that expression is responsive to environmental Fe bioavailability, showing low levels of expression under Fe repletion and robust mRNA and protein accumulation under Fe deficiency (**Figure 2B and 2C**). Using a reporter strain expressing a *SIT1*mCherry fusion gene from the endogenous *SIT1* locus, we ascertained whether internalized *C. glabrata* cells sense differences in genetically-programmed macrophage Fe status. As shown in **Figure 5A** by live microscopy, *C. glabrata* cells phagocytosed by macrophages expressing the *FPN^C326Y^* transgene showed enhanced Sit1mCherry accumulation when compared to yeast internalized by macrophages that were Fe-loaded by virtue of Fpn^H32R^ expression, suggesting that *C. glabrata* cells experienced different degrees of Fe deficiency in these macrophages. Fluorescein-conjugated dextran beads were included in the infection medium to allow for a clearer visualization of intra- versus extra-cellular yeast. To quantitatively evaluate the influence of macrophage Fe status on the Fe satiety of *C. glabrata*, *SIT1* transcript levels were monitored in yeast cells recovered from macrophages by semi-quantitative RT-PCR. **Figure 5B** shows that cells recovered from *FPN^H32R^*-expressing macrophages exhibited lower levels of the *SIT1* transcript when compared to cells recovered from *FPN^C326Y^-*expressing macrophages two and four hours post-infection, consistent with an increased Fe satiety within the phagosome. These results support the hypothesis that chronic perturbations in macrophage Fe levels translate into altered microbial Fe availability that may impact on the mechanisms of microbial Fe uptake.

### *Ex-vivo* infection of mouse primary macrophages supports a critical role for macrophage Fe loading on the dependence of *C. glabrata* on Sit1-mediated siderophore utilization

**Figure 6:**
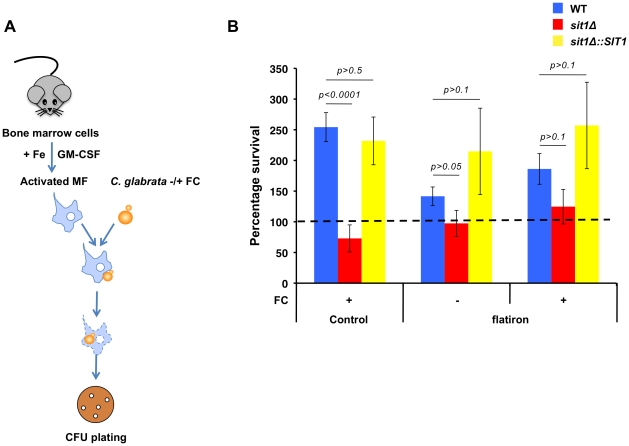
*C. glabrata* shows Sit1-dependent siderophore utilization in primary macrophages derived from wild type but not the *flatiron* mouse. **A** Schematic flowchart for infection of primary mouse macrophages with *C. glabrata*. Primary macrophages were differentiated from bone marrow cells harvested from control and *flatiron* mice, infected with the wild type, *sit1*Δ and reconstituted *sit1*Δ*::SIT1 C. glabrata* strains and CFU quantitated. Fe was added to the macrophage culture medium to stimulate *FPN* expression and cells were washed 3 times prior to infection with yeast strains. Data were analyzed with use of one-way ANOVA. **B** Infection of control primary bone marrow macrophages with *C. glabrata* showed robust Sit1-dependent survival with ferrichrome supplementation when compared to wild type *C. glabrata* grown exclusively under Fe deficiency (dashed line set at 100). This siderophore-dependent enhanced survival was absent in *C. glabrata* cells phagocytosed by primary macrophages derived from the *flatiron* mouse. Each experiment included 6 replicates per experimental sample. Results are shown as mean survival compared to the wild type grown in the absence of ferrichrome supplementation and are representative of at least 3 experiments. Bars represent the standard error.

Given the effects of macrophage Fe status on the dependence of *C. glabrata* siderophore utilization using well-established macrophage cell lines, we ascertained whether this could be recapitulated in an *ex-vivo* infection of primary mouse macrophages. Bone marrow cells were recovered from the femurs of control mice and of the *flatiron* (*ffe*) mouse model of human Ferroportin Disease, which harbors the *FPN^H32R^* mutation [36]. Cells were differentiated into mature macrophages in culture and endogenous Fpn expression stimulated by incubating cells in 10 µM ferric ammonium citrate for 2 days prior to activation and infection with *C. glabrata* (**Figure 6A**). Consistent with the results obtained using the *J774A.1* and U937 cell lines, when grown in the presence of ferrichrome, *C. glabrata* wild type and *SIT1*-reconstituted cells showed enhanced survival to the killing activities of wild type primary macrophages when compared to the *sit1*Δ strain (**Figure 6B**). However, siderophore-dependent survival was significantly diminished in infected *ffe* macrophages, supporting our previous observations that increased macrophage labile Fe pools promote the growth of *C. glabrata* independently of siderophore utilization.

### Integrity of the Sit1 SITD is critical for ferrichrome utilization

Computational analysis of the Sit1 SITD (**Figure 2A**) revealed a high degree of conservation among orthologous siderophore transporters in a large number of fungal species, including several animal and plant pathogens, a representative number of which are shown in **Figure S4**. To begin to evaluate a potential role for the SITD in Sit1-mediated siderophore utilization in *C. glabrata*, the predicted extracellular topological orientation of the carboxyl-terminal loop between transmembrane domains 13 and 14 was first evaluated experimentally. Sit1 was tagged with a 2XFLAG epitope within the carboxyl-terminal loop between residues G542 and D543 (**Figure S5A**). Cells expressing this tagged Sit1 protein, or the empty vector, were cultured under Fe deficient conditions and the accessibility of the anti-Flag antibody for its cognate epitope evaluated in non-permeabilized cells by indirect immunofluorescence. As shown in **Figure S5B**, plasma membrane accumulation of the Flag antibody was observed in non-permeabilized cells expressing carboxyl-loop-Flag Sit1. This immunofluorescence signal was absent in cells expressing vector alone and is consistent with an orientation of this loop region, and therefore the SITD, toward the extracellular face of the plasma membrane.

**Figure 7:**
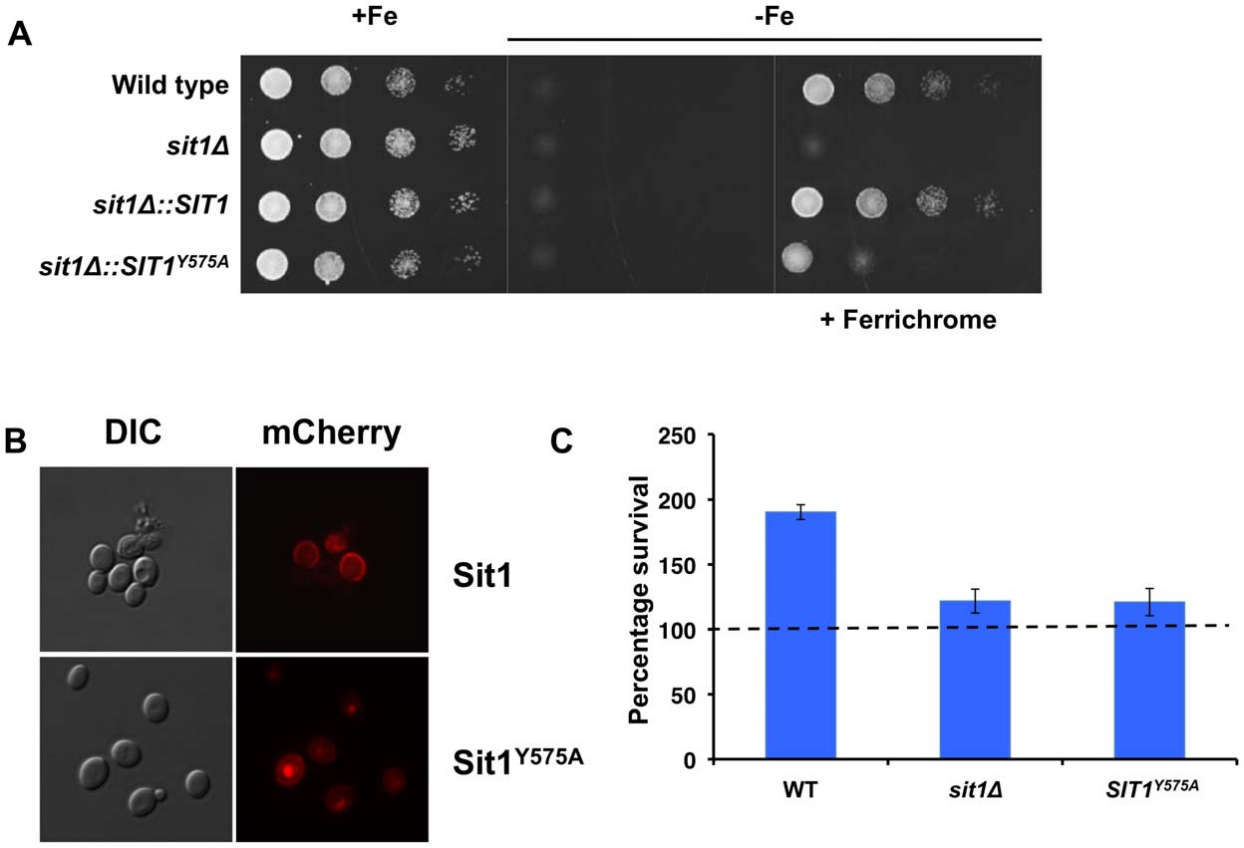
A conserved residue in the Sit1 SITD compromises ferrichrome utilization and survival in macrophages. **A** The Sit1^Y575A^ strain is compromised in ferrichrome utilization under Fe deficiency. *C. glabrata* strains were grown to mid-logarithmic phase and 10-fold serial dilutions spotted onto rich synthetic media and Fe-deficient media supplemented or not with 10 µM ferrichrome. **B** Sit1^Y575A^ localizes to the plasma membrane. In vivo fluorescence of wild type Sit1mCherry and Sit1^Y575A^mCherry strains cultured under Fe deficiency. **C** The Sit1^Y575A^ strain is compromised the ability to use siderophores to survive macrophage killing. Activated *J774A.1* cells were infected with wild type, *sit1*Δ or *sit1*Δ*::SIT1^Y575A^ C. glabrata* strains and CFU quantitated. Results are shown as survival relative to wild type *C. glabrata* grown under Fe deficiency represented by a dashed line. Each experiment included 6 replicates per experimental sample. Results are shown as mean survival compared to the wild type grown in the absence of ferrichrome supplementation and are representative of at least 3 experiments. Bars represent the standard error.

To test whether the Sit1 SITD may be of functional importance, we mutated the conserved residue, Y575 to alanine (**Figure S4**), and tested whether this might compromise Sit1-mediated ferrichrome utilization. A strain carrying the *SIT1^Y575A^* allele integrated at the endogenous genomic locus (**Figure S5C**) was strongly impaired in its ability to use ferrichrome as an Fe source (**Figure 7A**) despite exhibiting levels of protein expression (**Figure S5D**) and plasma membrane localization (**Figure 7B** and **Figure S5E**) comparable to the wild type strain. The observed decrease in the ability of the Sit1^Y575A^ mutant to utilize ferrichrome prompted us to evaluate the ability of this strain to survive macrophage killing when grown in the presence of this siderophore. In contrast to wild type *C. glabrata* cells, neither the *sit1*Δ nor the strain bearing the Sit1^Y575A^ substitution in Sit1 showed the Sit1-mediated siderophore-dependent increase in the ability to survive macrophage killing, suggesting that although this mutation does not lead to a complete loss of Sit1 function (see **Figure 7A**), the remaining activity is insufficient to meet the cellular demand for Fe within the phagosome (**Figure 7C**).

## Discussion

*Candida glabrata* has risen from relative obscurity as a relevant opportunistic human fungal pathogen to an increasing cause of serious infectious disease particularly in immunocompromised individuals. Yet, the significant phylogenetic relatedness of *C. glabrata* to *Saccharomyces cerevisiae* argues for some fundamental differences in the biology of this microorganism, either through the functions of the as yet poorly studied 400 genes unique to *Candida glabrata* and/or through the enhancement or acquisition of novel functions of shared genes. Our results underscore a role for siderophore utilization in the successful existence of *C. glabrata* as a human commensal as well as during pathogenesis, primarily with respect to survival to phagocytosis by mammalian macrophages. Furthermore, our studies support a pivotal contribution for unregulated host Fe acquisition in the outcome of fungal infection.

The severe Fe restriction imposed by mammalian hosts as an antimicrobial strategy dictates that Fe acquisition is a bona fide virulence determinant. The existence of clinical correlations between elevated Fe bioavailability and increased microbial burden extend beyond diseases of Fe overload for which the higher susceptibility to bacterial infection, notably to *Vibrio vulnificus*, has been described [15], [18]. In the same way, transfusion-dependent thalassemia patients present with increased susceptibility to widespread microbial infection that correlates with the degree of Fe overload [19]. Treatment of HH and thalassemia patients is further complicated by the fact that the sole FDA-approved Fe chelator desferrioxamine, a naturally occurring bacterial siderophore, effectively further solubilises Fe in a form that is readily utilized by a diversity of bacterial and fungal species, thus elevating the potential for infectious disease in these patients. Individuals with malignant hematological disorders, such as acute myeloid leukemia, who typically present with very high serum Fe, serum ferritin and transferrin saturation, are known to sustain increased mycoses, especially by *Candida* and *Aspergilli* [38], [39]. Furthermore, clinical evidence also indicates that Fe overload is an independent risk factor for post-transplant infection [20], [21] and correlates with poor prognosis and in patients infected with HIV, a virus known to depend on and manipulate target cellular Fe accumulation [40]. What then are the implications of Fe overload diseases to the susceptibility to fungal infections? Previous studies support the notion that optimal immunity operates within a range of physiological Fe concentrations [41]. This is ensured in part through Fpn activity, mobilizing dietary and systemic Fe, and the regulatory action of hepcidin. Hereditary Hemochromatosis presents with different genetic etiologies that impart distinct effects on macrophage Fe levels. Using mutations associated with Ferroportin Disease, an autosomal dominant disease in humans, we provide evidence that *C. glabrata* dependence on the siderophore-Fe uptake machinery is inversely correlated to labile macrophage Fe levels and that Fe overloaded macrophages are markedly less efficient at killing and containing *C. glabrata.* We infer that this results from the inability of Fe-laden macrophages to withhold this essential metal from internalized *C. glabrata* cells given the decreased dependence of *C. glabrata* on siderophore-Fe, decreased *SIT1* expression indicative of increased Fe satiety, increased *C. glabrata* fitness and the increased levels of host cell ferritin expression. Previous studies have shown that activated macrophages accumulate Cu within the phagosome [42] suggesting perhaps that inefficiently withheld macrophage Fe can be mobilized through reductive Fe uptake and supporting a host-dependent modulation of high-affinity mechanisms of Fe assimilation. This notion of a host modulatory action over the mechanisms utilized for microbial Fe acquisition is further evidenced in a study by Lesuisse [43] that compared the effects of exposure of *C. albicans* to either fetal bovine serum or synthetic Fe-deficient medium supplemented with distinct Fe sources. The results obtained showed that reductive Fe uptake from ferric citrate was downregulated in the presence of serum, as evidenced by decreased Fe uptake and reduced ferrireductase activity, whereas that from ferrichrome-type siderophores was elevated.

In contrast to Fe-loaded macrophages, the low macrophage Fe levels associated with Fpn^C326Y^ expression impose Fe deficiency on phagocytosed *C. glabrata* cells, as demonstrated by elevated *SIT1* expression and further supported by the increased dependence on ferrichrome and Sit1 for proliferation. As a result of chronic Fe-deficiency, hepcidin-insensitive macrophages are expected to represent a formidable challenge for microbial proliferation. The *FPN^C326Y^* disease-associated mutation results in a similar clinical outcome to that observed in patients carrying *HFE* mutations present in approximately 80% of HH patients and exhibiting an allelic frequency of over 10% in Caucasians of European descent [13]. Given the high prevalence of *HFE* mutations, it has been postulated that this mutation was positively selected for conferring protection during outbreaks of infections caused by intracellular pathogens such as *Chlamydia* and *Yersinia* [44], [45] highlighting the intimate association between host Fe status and infectious disease.

Whilst siderophore utilization is also undoubtedly critical to microbial colonization of healthy immunocompetent individuals in its state of commensalism, our study prompts an as yet unresolved question in the field regarding the origin of xenosiderophore substrates that are utilized by non-siderophore producers such as *Candida* and *Cryptococcus* species. A recent study by Ghannoum et al. characterizing the mycobiome of the oral cavity of healthy individuals identified in this host milieu a complex and dynamic profile of fungal species, the number of which parallels that observed for bacteria [46]. Among the fungi found to colonize the oral cavity of humans are several siderophore-producers that include *Aspergillus* and *Fusarium* species known to copiously produce siderophores that include coprogens and ferrichromes, both substrates for Sit1-facilitated utilization in *C. glabrata*. In particular, *Aspergillus fumigatus*, one of the most prevalent air-borne human pathogens, synthesizes chemically distinct siderophores for the solubilization of extracellular Fe as well as for its safe intracellular storage [47]. In fact, siderophore utilization by this saprophytic fungus has been shown to be required for virulence, in contrast to reductive Fe assimilation [48], and the conidia of a siderophore-deficient mutant exhibit decreased levels of stored Fe and higher sensitivity to oxidative stress [47]. Given the ubiquitous nature of siderophore production and the mixed nature of the mammalian microbiome localized siderophore reservoirs are likely available to *C. glabrata* and other non-siderophore producers. In particular, it is tempting to speculate that the higher microbial burden associated with immunocompromised patients generates higher concentrations of microbial siderophores that might tip the balance towards the transition from commensalism to pathological *C. glabrata* proliferation and subsequent infectious disease.

Given the inability of *C. glabrata* to efficiently use heme, Sit1-dependent siderophore utilization represents a likely mechanism for high-affinity Fe uptake under conditions of Cu insufficiency, conditions under which elemental high affinity Fe uptake is compromised. This suggests that siderophore utilization might contribute toward the virulence of *C. glabrata* in both the Fe- and Cu-restricted mammalian host [49]. Sit1 is syntenic to its orthologous gene in *S. cerevisiae*, Arn1, with which it shares high sequence identity that translates into similar siderophore substrate specificity, which includes ferrichromes and coprogen. Yet, Sit1 and Arn1 differ in their subcellular localization. The routing of trans-Golgi Arn1 away from the plasma membrane and to the vacuolar protein-sorting pathway requires the monomeric clathrin adaptor, Gga2 [50]. Given the existence of a putative orthologue for Gga2 in *C. glabrata*, it is unclear whether differences in transporter localization reflect distinct protein-protein interactions or another mechanism of siderophore transport altogether - pore versus receptor, for instance. Interestingly, *C. albicans* Sit1 as well as S. cerevisiae Enb1 [7], also localize predominantly to the plasma membrane in either the absence or presence of substrate siderophore [51]. It is tempting to speculate that under the pressure of harsh competition for Fe and local xenosiderophore reservoirs, *C. glabrata* and *C. albicans* have evolved to become more competitive for siderophore binding by expressing the transporter constitutively at the cell surface. Further studies are required to elucidate the mechanism of siderophore recognition and uptake by Sit1 as well as its intracellular fate, an aspect of siderophore utilization that is currently poorly understood in fungi.

## Materials and Methods

### Yeast strains and growth conditions

*C. glabrata* strains, BG2 and BG14 (*ura3::KAN*), were a gift from Dr. Brendan Cormack at the Johns Hopkins School of Medicine, Maryland. Growth under Fe-replete conditions was achieved by supplementing media with 5 µM ferrous ammonium sulfate. Fe-deficient media contained 100 µM bathophenantroline disulphonate (BPS). For spot assays, cells were grown in synthetic complete medium (SC) to mid-logarithmic phase and serial dilutions were spotted on SC or SC supplemented with 100 µM BPS or 150 µM bathocuproine sulfonate (BCS) with or without the indicated siderophores. Ferrichrome, desferrioxamine, hemin and zinc protophorphyrin were purchased from Sigma Aldrich, St. Louis, MO; ferrirubin, coprogen, enterobactin, salmochelin, triacylfusarine C and yersiniabactin were purchased from EMC Microcollections, Tuebingen, Germany. Alexa Fluor-conjugated dextran was purchased from Invitrogen, Carlsbad, CA. For macrophage infection experiments, yeast cells cultured in low Fe medium for 5 hours were exposed or not to ferrichrome [10 µM] 30–35 minutes and the yeast cultures were washed three times with PBS prior to infection. The BG14 strain was used to generate the *sit1*Δ strain by the PRODIGE PCR-based approach [52]. The *SIT1FLAG* knock-in cassette was created by overlap PCR of the *SIT1* ORF and 964 bp upstream promoter was used to generate the in-frame *SIT1FLAG*-fusion DNA subcloned upstream of the nourseothricin resistance (*NAT^R^*) cassette with a further 400 bp of the *SIT1* 3'UTR cloned downstream of *NAT^R^*. The *sit1*Δ*::SIT1mCherry* strain was generated by targeted integration of a *SIT1mCherryNAT^R^* cassette cloned into pBM46. Generation of *SIT1* tagged with two FLAG epitopes between residues G542 and D543 within the carboxyl-terminal loop was generated by overlap PCR. The Sit1Y575A mutation was generated by site-directed mutagenesis (QuikChange Site-Directed Mutagenesis Kit, Stratagene, La Jolla, CA) and either integrated at the endogenous genomic locus or expressed episomally. For *C. glabrata* transformation, overnight cultures were transformed with 5 µg DNA using the Frozen-EZ Yeast Transformation II Kit (Zymo Research, Orange, CA) according to the manufacturers instructions. Transformants were selected by growth on YPD media containing 200 µg/ml nourseothricin (Werner BioAgents, Jena, Germany) at 30 °C for 2–4 days.

### RNA and protein analyses

Cells were cultured in SC containing ferrous ammonium sulfate [5 µM] in the absence or presence of BPS [80 µM] and samples harvested at the indicated time points and processed for RNA and protein extraction. Total RNA was extracted using the hot acid phenol method [53]. PCR-amplified probes were gel-purified and radiolabeled with alpha-P^32^-dCTP (GE Healthcare, Piscataway, NJ) using Random Primed DNA Labeling Kit (Roche Applied Science, Indianapolis, IN). Semi-quantitative PCR was performed on cDNA generated by reverse transcription of isolated total RNA (Superscript III first strand; Invitrogen, Carlsbad, CA) using primers specific for *SIT1* and *ACT1*. *SIT1* expression levels were normalized against those of *ACT1*. Total protein was extracted in ice-cold lysis buffer (Tris 25 mM, pH7.5, NaCl 150 mM, 1 mM EDTA) supplemented with protease inhibitors (Roche complete tablet; Roche Applied Science, Indianapolis, IN) using the Triton X-100/glass bead method and total protein quantified using BCA reagent (Pierce, Rockford, IL). Sit1Flag was detected using an HRP-conjugated α-Flag antibody (Sigma Aldrich, St. Louis, MO). An alpha-Pgk1 antibody was used as loading control.

### Fluorescence and indirect immunofluorescence microscopy

The *sit1*Δ*::SIT1mCherry* cells were visualized with a Zeiss Axio Image widefield fluorescence microscope. In vivo visualization of macrophages infected with *C. glabrata* strain *sit1*Δ*::SIT1mCherry* was performed using a Zeiss Axio Observer Z1 fluorescence microscope. Indirect immunofluorescence of Fpn was observed and captured using a motorized Zeiss Axio Observer Z1. Cells were fixed in methanol at −20 °C for 5 minutes, incubated with Fpn antibody (a gift from Drs. Jerry Kaplan and Ivana de Domenico, University of Utah) and incubated with a secondary fluorochrome-conjugated antibody (Alexa-fluor 488; Molecular Probes, Invitrogen, Carlsbad, CA).

### ROS measurement

*J774A.1* macrophages seeded at 2×10^5^ cells/ml were activated with 1 µg/ml LPS and 5 ng/ml LPS for 3 hours and 2 µM 2′,7 dichlorofluorescein diacetate (DCFH-DA) added during the last 15 minutes. Fluorescence was monitored 450 nm excitation and 530 nm emission using a fluorescence plate reader (Perkin-Elmer 1420 Victor 3).

### Mammalian cell infection assay

The mouse macrophage cell line *J774A.1* was maintained in Dulbecco's Modified Eagles Medium 4.5 g/L D-glucose (Gibco, Invitrogen, Carlsbad, CA), 10% FBS, 100U penicillin and 100 mg/ml streptomycin, at 37 °C and 5% CO_2_. For iron loading experiments, *J774A.1* macrophages were cultured in 20 µM ferric ammonium citrate for 2 days and washed 3 times with warm DMEM prior to infection with *C. glabrata*. Macrophages seeded at 2×10^5^ cells/ml overnight were activated with IFN-gamma [5 ng/ml] (Sigma Aldrich, St. Louis, MO) and LPS [1 µg/ml] (Sigma Aldrich, St. Louis, MO) 3 hours prior to infection with *C. glabrata* strains at a multiplicity of infection of 1:4 (macrophage:yeast). At the indicated time points infected macrophages were lysed with water, and dilutions of the lysates plated on YPD. Colony forming units (CFU) were counted after growth at 30 °C for 2 days. U937 cells were maintained in RPMI-1640 medium (Gibco, Invitrogen, Carlsbad, CA), supplemented with 2 mM glutamine, 10% FBS, 100U penicillin and 100 mg/ml streptomycin, at 37 °C and 5% CO_2_. Differentiation of these pro-monocytic cells into mature macrophages was induced by the addition of 10 nM phorbol myristate acetate (PMA) to 1×10^6^ cells/ml for 48 hours. Cells were washed 3 times with warm culture medium and incubated for a further 48 hours at 37 °C and 5% CO_2_. LPS (1 µg/ml) was added to the macrophages 3 hours prior to infection with *C. glabrata* (MOI of 1:1) and yeast cell recovery and CFU counting performed as described above. For primary macrophage infection assays, mouse femurs were kindly supplied to us by Drs. Jerry Kaplan and Ivana De Domenico, at the University of Utah. Primary bone marrow cells were harvested by flushing the femurs of 3–5 month-old female control (C3H) or *flatiron* (*ffe)* mice and macrophage differentiation induced by culturing in RPMI 1640 containing 20% FBS, 20 µM ß-mercaptoethanol, 2 mM L-glutamine, 100U penicillin and 100 mg/ml streptomycin and GM-CSF [3 ng/ml]. Macrophages were seeded for infection assays at 2×10^5^ cells/ml.

For the generation of stable cell lines expressing wild type and mutant forms of *FPN*, full-length mouse *FPN* cDNA (TrueORF, OriGene, Rockville, MD) was used as a template to introduce the H32R and C326Y mutations through site-directed mutagenesis. *FPN*, *FPN^H32R^* and *FPN^C326Y^* plasmid DNA were stably transfected into *J774A.1* macrophages using Lipofectamine reagent (Invitrogen, Carlsbad, CA). Total protein was extracted in lysis buffer as described above supplemented with 0.1% SDS and a phosphatase inhibitor cocktail (HALT, Pierce, Rockford, IL). Antibodies against ferritin and α-tubulin were purchased from Abcam (Cambridge, MA). IL-6 and TNF-alpha secretion was measured by enzyme-linked immunosorbent assay (ELISA) (R&D Systems, Minneapolis, MN). Fluorescence was measured using a SpectraMax Plus luminometer (Molecular Devices, Sunnyvale, CA).

### Accession numbers

The *SIT1* DNA, protein sequence and functional description were deposited in GenBank with the Accession Number HQ734814.

## Supporting Information

Figure S1 Complementation assays showing the functionality of the *C. glabrata* Sit1 Sit1mCherry and Sit1Flag fusion proteins. A. Alignment of *C. glabrata* Sit1 and *S. cerevisiae* Arn1 protein sequences. The protein sequences of *S. cerevisiae* Arn1 and *C. glabrata* Sit1 were aligned using ClustalW. B. The Sit1mCherry and Sit1Flag fusion proteins are functional for siderophore utilization. The fusion genes were each independently integrated back into the *sit1* Δ strain at the endogenous genomic locus and functionality confirmed by growth on low Fe media supplemented with ferrichrome. C. Sit1 is unable to facilitate the utilization of the bacterial siderophores desferrioxamine (DFO), enterobactin (EB), salmochelin (S4) or yersiniabactin (YB) or the fungal siderophore triacetylfusarine C (TAFC). *C. glabrata* strains were grown to mid-logarithmic phase and serial dilutions were spotted onto rich synthetic media and Fe-deficient media supplemented or not with the indicated siderophores. Ferrichrome was used at a concentration of 10 µM and BPS was added at a concentration of 100 µM. All other siderophores were used at a concentration of 50 µM. D. Plasma membrane localization of Sit1mCherry using in vivo immunofluorescence in response to Fe deficiency in the presence or absence of ferrichrome.
(2.67 MB TIF)

Figure S2 *C. glabrata* survival kinetics and characterization of activated mouse macrophages. A. Kinetics of *C. glabrata* survival within activated macrophages in the presence or absence of ferrichrome. Infection of macrophages with *C. glabrata* wild type, *sit1* Δ and *SIT1*-reconstituted strains was performed as described in Materials and Methods. *C. glabrata* cells were recovered at the indicated time-points and CFU determined. B. Culturing macrophages in high exogenous Fe conditions leads to an increase in intracellular Fe stores. Macrophages were cultured for 2 days in the presence or absence of Fe supplementation in the culture medium. Cells were harvested and processed for the isolation of total protein. C. Macrophage Fe-loading does not lead to an overt defect in activation as evaluated by TNF-α secretion. *J774A.1* macrophages were grown in the presence or absence of 10 µM ferric ammonium citrate for 2 days and activated with 5 ng/ml IFN-γ and 1 µg/ml LPS for 3 hours. The supernatant was collected and processed for ELISA. D. The generation of reactive oxidative species was quantified by monitoring the conversion of DCFH-DA into the highly fluorescent DCF. Macrophages were either untreated (control) or exposed to LPS/IFN-γ or menadione (250 µM) for 3 hours and DCFH-DA (20 µM) added to the samples during the last 30 minutes. Absorbance was read at 520 nm. The results shown are mean fluorescence (AU) of 8 replicates per sample and the bars show the standard error of the mean.
(0.47 MB TIF)

Figure S3 Characterization of the Fpn cell lines. A. Fpn localization in macrophage stable cell lines expressing wild type and mutant forms of Fpn. Indirect immunofluoresence was used to evaluate the localization of Fpn in the presence or absence of hepcidin. White arrows indicate the cell surface localization of Fpn most evident on psudopod formations. B. Macrophage stable cell lines expressing wild type and mutant forms of Fpn are capable of mounting an inflammatory response. Supernatants from stable cell lines expressing wild type or ferroportin mutation alleles were treated as described above and the supernatants collected for ELISA. Control - non-transfected *J774A.1* cells.
(1.34 MB TIF)

Figure S4 Identification of the Sit1 SITD. Representative BLAST results retrieved using a 51-residue sequence comprising the predicted carboxyl-terminal loop. Representative set of the 91 hits obtained is shown. Mutation of Sit1^Y575A^ was performed using site-directed mutagenesis.
(2.14 MB TIF)

Figure S5 The integrity of the Sit1 carboxyl-terminal loop is critical for siderophore utilization. A. Schematic illustration of the localization of the Flag epitope introduced within the carboxyl-terminal loop. B. Sit1-SITDFlag localizes to the plasma membrane in non-permeabilized cells. Cells carrying the Sit1-SITDFlag construct of vector alone were grown under Fe deficiency and subjected to indirect immunofluorescence using anti-Flag antibody. C. Schematic illustration of the Sit1^Y575A^ substitution introduced into the SIT1MCHERRY construct and integrated at the *SIT1* endogenous locus. D. Sit1 protein levels of the wild type and Sit1^Y575A^ strains under Fe deficiency or Fe sufficiency. Cells were grown either under Fe deficiency by supplementation of BPS to the growth medium or Fe sufficiency by the further supplementation of ferrichrome for 3 hours. Cells were harvested and processed for protein analysis. E Subcellular localization of the Sit1^Y575A^Flag mutant protein. The mutant transporter was expressed episomally and its subcellular localization evaluated by indirect immunoflourescence of the Flag epitope.
(1.30 MB TIF)
