# Supplementary figures and images for: Host Iron Withholding Demands Siderophore Utilization for Candida glabrata to Survive Macrophage Killing
Source: PLoS Pathog. 2011 Mar 17;7(3):e1001322. doi: 10.1371/journal.ppat.1001322 (PMC3060170; doi:10.1371/journal.ppat.1001322)

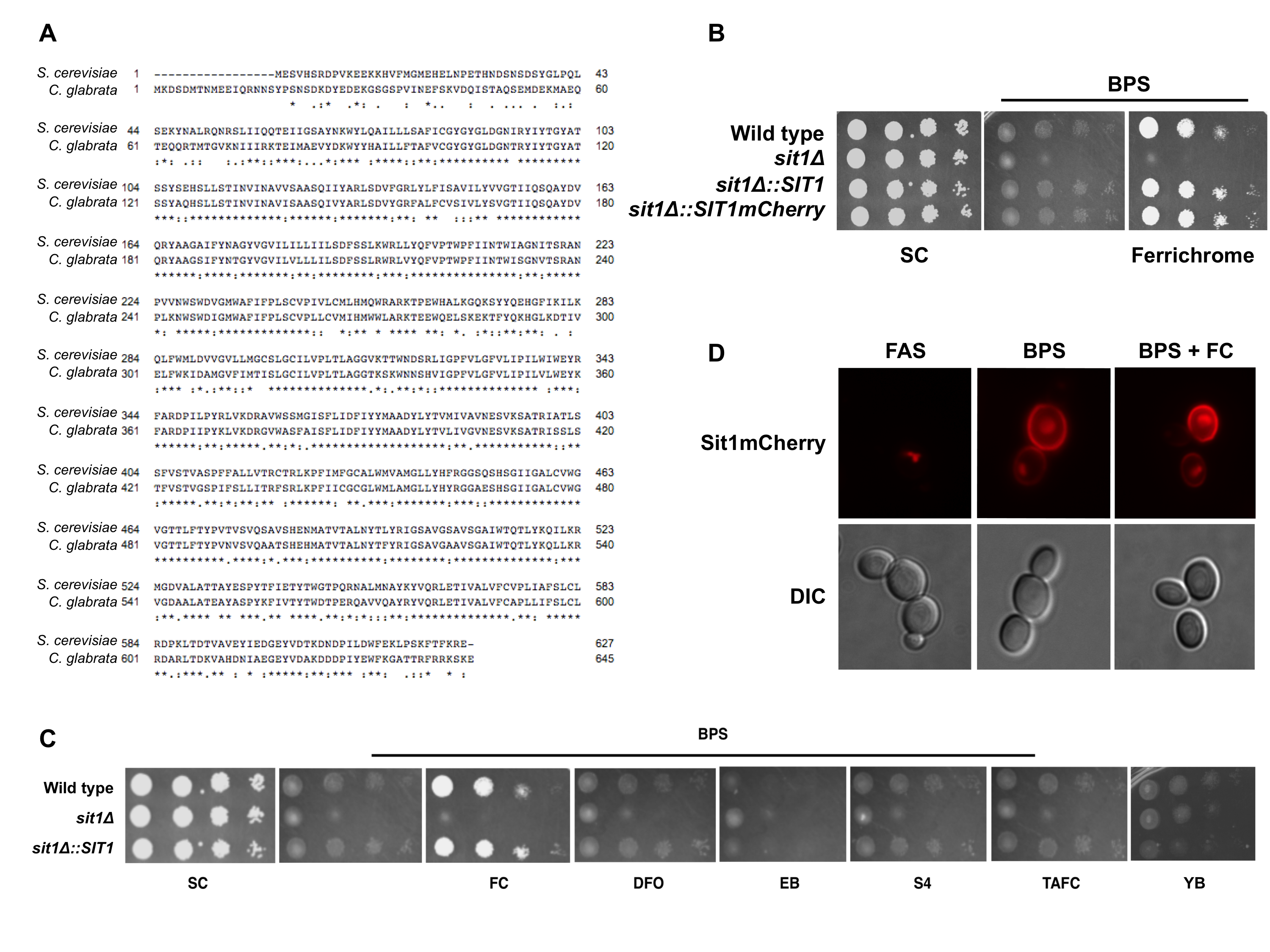

Supplement: Figure S1 — Complementation assays showing the functionality of the C. glabrata Sit1 Sit1mCherry and Sit1Flag fusion proteins. A. Alignment of C. glabrata Sit1 and S. cerevisiae Arn1 protein sequences. The protein sequences of S. cerevisiae Arn1 and C. glabrata Sit1 were aligned using ClustalW. B. The Sit1mCherry and Sit1Flag fusion proteins are functional for siderophore utilization. The fusion genes were each independently integrated back into the sit1 Δ strain at the endogenous genomic locus and functionality confirmed by growth on low Fe media supplemented with ferrichrome. C. Sit1 is unable to facilitate the utilization of the bacterial siderophores desferrioxamine (DFO), enterobactin (EB), salmochelin (S4) or yersiniabactin (YB) or the fungal siderophore triacetylfusarine C (TAFC). C. glabrata strains were grown to mid-logarithmic phase and serial dilutions were spotted onto rich synthetic media and Fe-deficient media supplemented or not with the indicated siderophores. Ferrichrome was used at a concentration of 10 µM and BPS was added at a concentration of 100 µM. All other siderophores were used at a concentration of 50 µM. D. Plasma membrane localization of Sit1mCherry using in vivo immunofluorescence in response to Fe deficiency in the presence or absence of ferrichrome. (2.67 MB TIF) [file ppat.1001322.s001.tif]

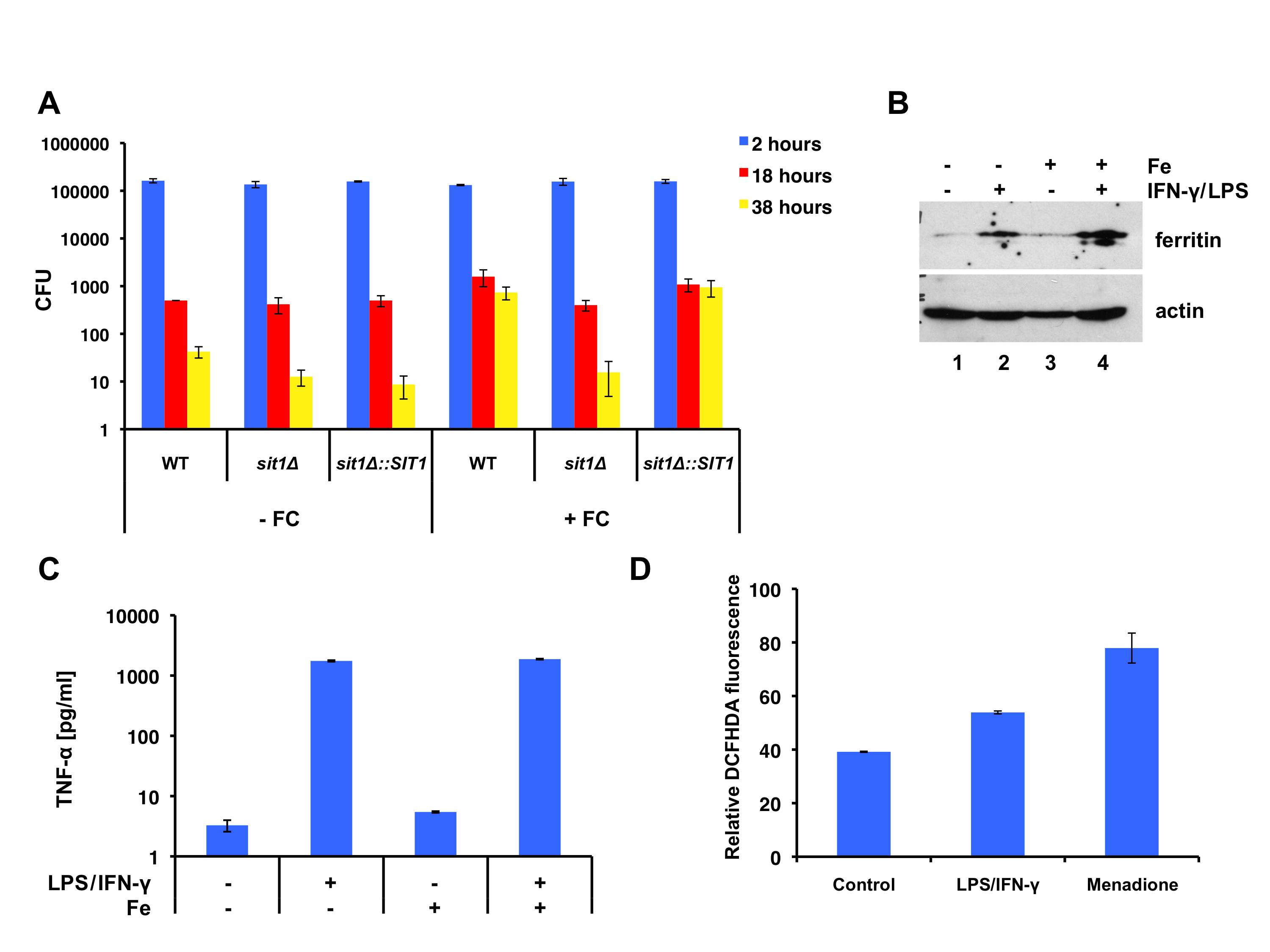

Supplement: Figure S2 — C. glabrata survival kinetics and characterization of activated mouse macrophages. A. Kinetics of C. glabrata survival within activated macrophages in the presence or absence of ferrichrome. Infection of macrophages with C. glabrata wild type, sit1 Δ and SIT1-reconstituted strains was performed as described in Materials and Methods. C. glabrata cells were recovered at the indicated time-points and CFU determined. B. Culturing macrophages in high exogenous Fe conditions leads to an increase in intracellular Fe stores. Macrophages were cultured for 2 days in the presence or absence of Fe supplementation in the culture medium. Cells were harvested and processed for the isolation of total protein. C. Macrophage Fe-loading does not lead to an overt defect in activation as evaluated by TNF-α secretion. J774A.1 macrophages were grown in the presence or absence of 10 µM ferric ammonium citrate for 2 days and activated with 5 ng/ml IFN-γ and 1 µg/ml LPS for 3 hours. The supernatant was collected and processed for ELISA. D. The generation of reactive oxidative species was quantified by monitoring the conversion of DCFH-DA into the highly fluorescent DCF. Macrophages were either untreated (control) or exposed to LPS/IFN-γ or menadione (250 µM) for 3 hours and DCFH-DA (20 µM) added to the samples during the last 30 minutes. Absorbance was read at 520 nm. The results shown are mean fluorescence (AU) of 8 replicates per sample and the bars show the standard error of the mean. (0.47 MB TIF) [file ppat.1001322.s002.tif]

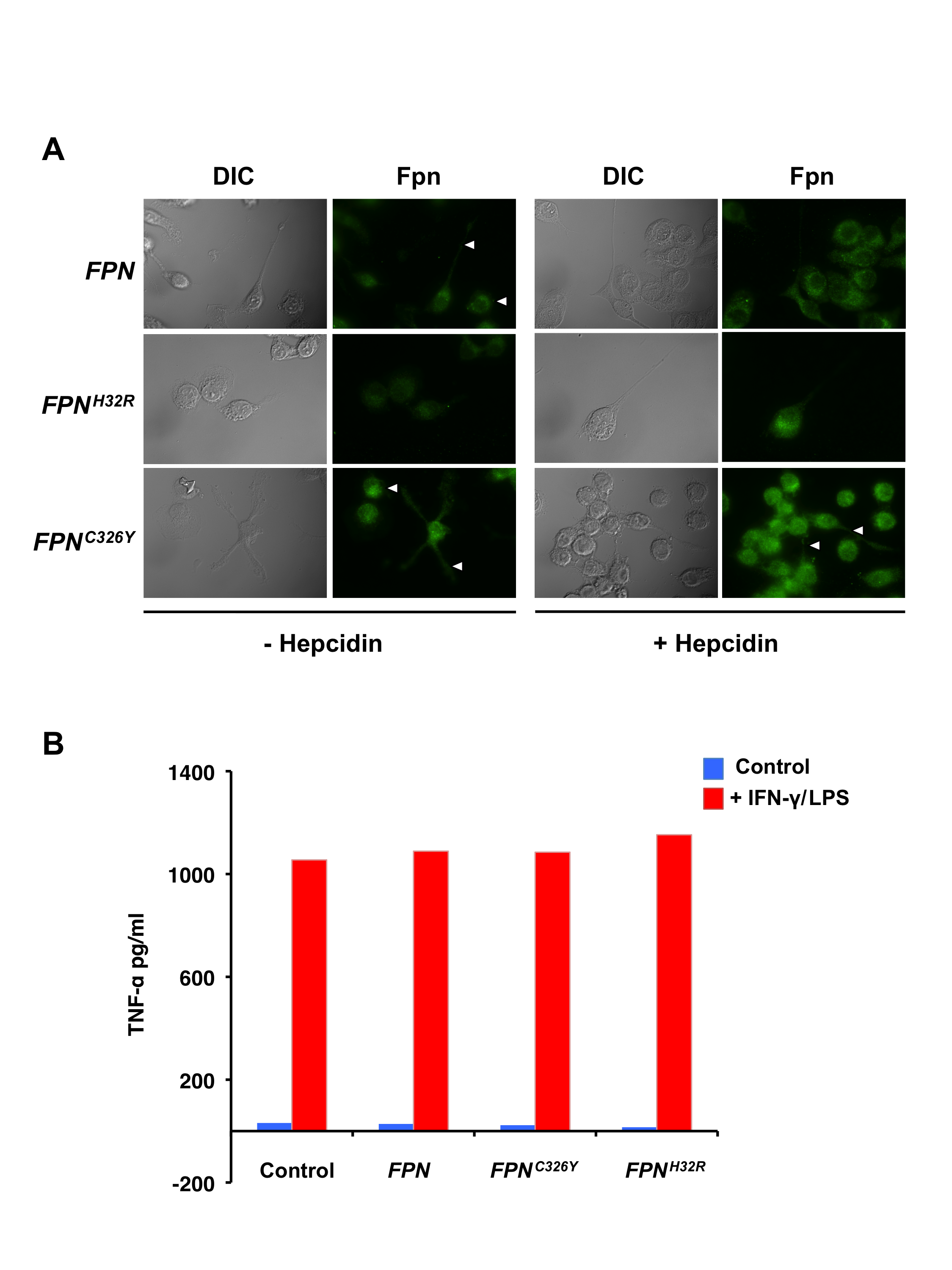

Supplement: Figure S3 — Characterization of the Fpn cell lines. A. Fpn localization in macrophage stable cell lines expressing wild type and mutant forms of Fpn. Indirect immunofluoresence was used to evaluate the localization of Fpn in the presence or absence of hepcidin. White arrows indicate the cell surface localization of Fpn most evident on psudopod formations. B. Macrophage stable cell lines expressing wild type and mutant forms of Fpn are capable of mounting an inflammatory response. Supernatants from stable cell lines expressing wild type or ferroportin mutation alleles were treated as described above and the supernatants collected for ELISA. Control - non-transfected J774A.1 cells. (1.34 MB TIF) [file ppat.1001322.s003.tif]

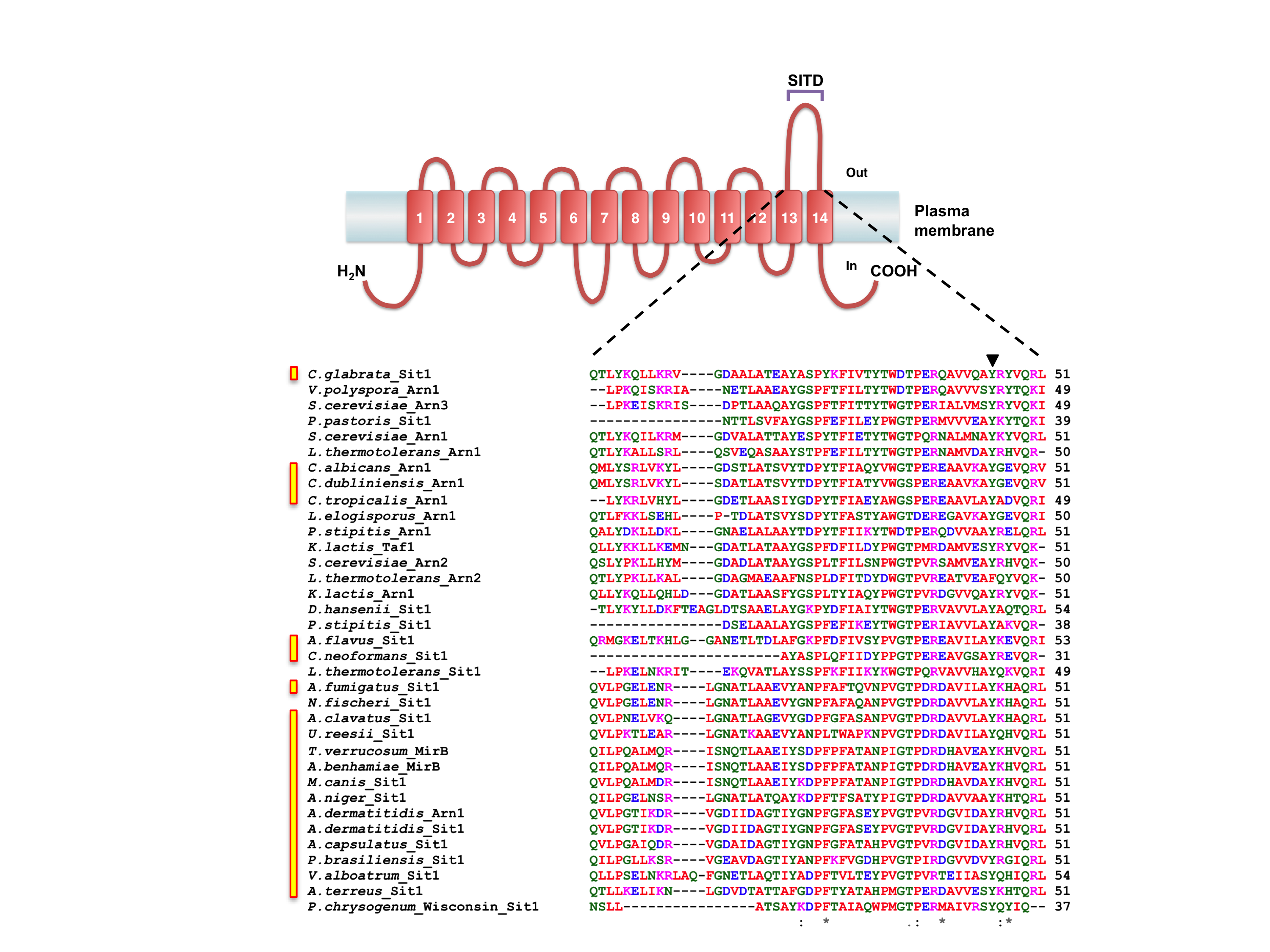

Supplement: Figure S4 — Identification of the Sit1 SITD. Representative BLAST results retrieved using a 51-residue sequence comprising the predicted carboxyl-terminal loop. Representative set of the 91 hits obtained is shown. Mutation of Sit1Y575A was performed using site-directed mutagenesis. (2.14 MB TIF) [file ppat.1001322.s004.tif]

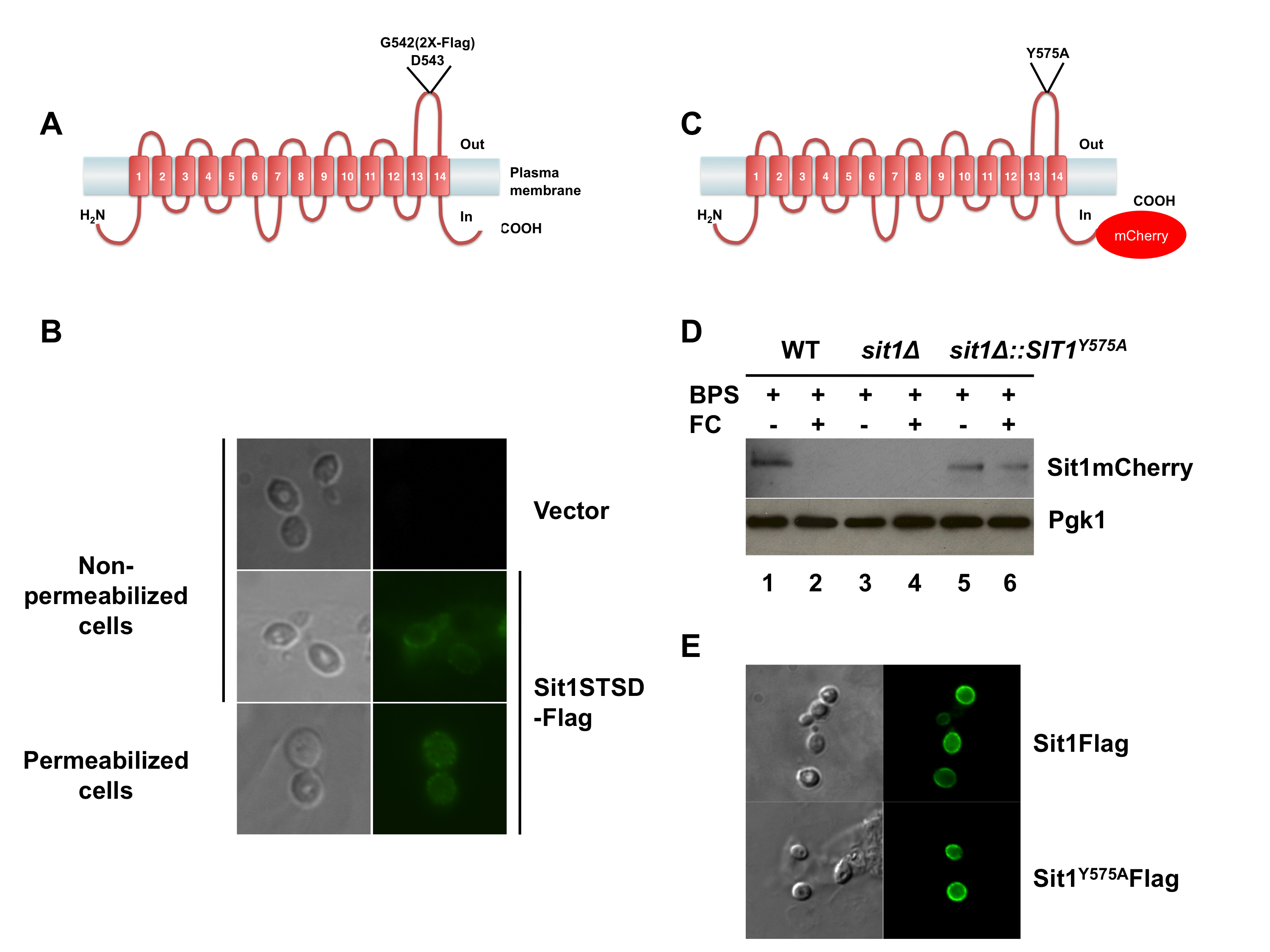

Supplement: Figure S5 — The integrity of the Sit1 carboxyl-terminal loop is critical for siderophore utilization. A. Schematic illustration of the localization of the Flag epitope introduced within the carboxyl-terminal loop. B. Sit1-SITDFlag localizes to the plasma membrane in non-permeabilized cells. Cells carrying the Sit1-SITDFlag construct of vector alone were grown under Fe deficiency and subjected to indirect immunofluorescence using anti-Flag antibody. C. Schematic illustration of the Sit1Y575A substitution introduced into the SIT1MCHERRY construct and integrated at the SIT1 endogenous locus. D. Sit1 protein levels of the wild type and Sit1Y575A strains under Fe deficiency or Fe sufficiency. Cells were grown either under Fe deficiency by supplementation of BPS to the growth medium or Fe sufficiency by the further supplementation of ferrichrome for 3 hours. Cells were harvested and processed for protein analysis. E Subcellular localization of the Sit1Y575AFlag mutant protein. The mutant transporter was expressed episomally and its subcellular localization evaluated by indirect immunoflourescence of the Flag epitope. (1.30 MB TIF) [file ppat.1001322.s005.tif]
